# The Structural Properties of Odorants Modulate Their Association to Human Odorant Binding Protein

**DOI:** 10.3390/biom11020145

**Published:** 2021-01-22

**Authors:** Tarsila G. Castro, Carla Silva, Teresa Matamá, Artur Cavaco-Paulo

**Affiliations:** Centre of Biological Engineering, University of Minho, Campus de Gualtar, 4710-057 Braga, Portugal; castro.tarsila@ceb.uminho.pt (T.G.C.); carla.silva@ceb.uminho.pt (C.S.); teresam@ceb.uminho.pt (T.M.)

**Keywords:** human odorant-binding protein, odorants, molecular dynamics simulations, molecular docking, virtual screening.

## Abstract

The binding of known odorant molecules to the human odorant-binding protein (hOBP) was evaluated *in silico.* Docking experiments elucidate the preferable binding site and binding affinity of odorant molecules to hOBP. The physicochemical properties molecular weight (MW), vapor pressure (Vp), hydrophobicity level (logP), number of double bonds (NºDB), degree of unsaturation (DoU) and the chemical classification, were selected for the study of odorant modulation. Here, these properties were analyzed concerning 30 pleasant and 30 unpleasant odorants, chosen to represent a wide variety of compounds and to determine their influence on the binding energy to hOBP. Our findings indicate that MW, logP and Vp are the most important odorant variables, directly correlated to odorant-binding energies (ΔG_binding_) towards hOBP. Understanding how the odorants behave when complexed with the OBP in human olfaction opens new possibilities for the development of future biotechnological applications, including sensory devices, medical diagnosis, among others.

## 1. Introduction

Olfaction functions as a chemosensing system, allowing the detection and discrimination of millions of different volatile molecules, the odorants, which provide extremely important information about the surrounding environment. In humans, odorants recognition is mediated by a large repertoire of olfactory receptors (ORs), coded by 391 functional OR genes [1]. The ORs are located across the plasma membranes of the ciliated dendrites of olfactory sensory neurons, localized in the olfactory epithelium. Each sensory neuron expresses a single allele of a single OR gene to ensure a distinct pattern of neuronal activation for every odorant [2]. Mammalian ORs belong to one of two classes, according to the recognized odorant type: Class I, ORs mostly bind hydrophilic odorants, and Class II, ORs bind hydrophobic odorants. In order for the ligand–receptor binding to occur, the odorant must cross a hydrophilic barrier—the mucus, where the ciliated dendrites of olfactory neurons are immersed. The hydrophobic odorants need to be transported, which is thought to be the role of the small soluble proteins, the odorant-binding proteins (OBPs).

In vertebrates, OBPs are highly expressed in the nasal epithelia, where they bind and carry, through the aqueous mucus, hydrophobic and volatile odorant molecules. Humans express two “classical” OBP genes, OBP2A and OBP2B, but, in contrast to other mammals, their expression in the human olfactory epithelium is not enhanced [1]. In vertebrates, OBP presents the typical folding of lipocalins, a hydrophobic β-barrel core that encloses an internal ligand-binding site. The obvious and crucial physiological role of OBP in olfaction is to facilitate the transport of odorant molecules to the ORs. Based on experimental evidence, an active role also has been proposed for OBPs in addition to the passive transport of odorants [2,3]. It was demonstrated in an in vitro assay that the rat OBP-F1 restores a OR activity at high odorant doses, changing the response curve from bell-shaped to sigmoidal, which is the characteristic curve of the OR in vivo response [3]. This active role was proposed to be mediated by a physical interaction between the OBP and the receptor, an interaction that is affected by the presence of the OR ligand. Furthermore, in another work, the authors have demonstrated that ORs constitutively form homodimers [4]. OR dimers displayed different conformational changes upon stimulation with various odorant doses, corresponding to different levels of activity. At low ligand concentrations, OR dimer would bind only one odorant molecule staying in an active form. While, at high ligand doses, the OR dimer would bind a second odorant molecule, leading to an inactive conformation. The authors assumed that in the presence of OBP, the second ligand would not be able to bind due to the OBP binding to OR dimer. In this way, the active role of OBP could be an allosteric control of OR dimer activity, at high ligand doses, besides the intrinsic and passive ability of OBP to buffer the levels of odorants.

OBP has been used in the biotechnology field for many purposes [5,6,7], as in the development of sensors [8] or smart textiles [9]. From a biochemical point of view, we have studied porcine OBP (pOBP) for its functionalization with small peptides, which promoted the liposomal transduction of a small molecule, as well as for the ability of a truncated form to have a thermal response [10,11]. These studies have shown the versatile profile of this protein under different conditions and environments.

Odorant molecules are volatile and structurally diverse compounds, which are perceived by the ORs, carrying information about the surrounding environment. The odorants can be detected directly from the inhaled air through the nose or by the throat, after chewing food. Yet, often, these molecules cannot cross the nasal mucus without support [12,13], which is the transport role of OBP.

The organoleptic classification of an odor substance should be understood loosely, i.e., the olfactive memory of each person, as well as the habituation to a specific aroma, may cause one to find an aroma more pleasant rather than other. That is why, herein, the odor compounds were categorized as generally pleasant or unpleasant, in contrast to terms like good/bad aromas. The selection of odors was thought to represent a wide variety of compounds in terms of the addressed physicochemical properties. In addition, we have prioritized the evaluation of aromas commonly used in industry or, in the case of unpleasant, body odors or decaying food smells.

Hence, far, the human OBP itself has been the subject of few studies [14], as the experimental characterization of its structure was only achieved in 2015. Recently, some works related an hOBP lysine to aldehyde odorants-binding [15,16]. These studies were conducted experimentally and by molecular dynamics (MD) simulations using an hOBP designed by homology modeling. Apart from that, the research in hOBP is still very incipient in contrast to OBPs from other vertebrates.

The present study is focused on the *in silico* analysis of human OBP (hOBP), aiming for a deeper understanding of the binding and transport of odorants by this protein while establishing a correlation between the type and strength of binding and the physicochemical properties of the odorants. Thus, a comprehensive library of 60 odorant compounds, 30 commonly perceived as pleasant and 30 as unpleasant, were analyzed regarding the binding affinity to the hOBP. These molecules were selected according to the established application in the industry (cosmetics and textiles) as well as to the structural characteristics, including size/volume, saturate/unsaturated bonds and functional groups. The functional groups included amines, alcohols, aldehydes, ketones, thiols, acids, phosphines, organosulfur compounds, esters, lactones and volatile gases, among others. The first binding studies in OBP were performed using heterocyclic and terpenoid derivatives and medium-size aliphatic alcohols and aldehydes [17,18]. The same rationale was used here; small to medium size odorant molecules were addressed. Docking virtual screening was applied to disclose the hOPB binding site, the interaction pattern and the affinity towards the 60 odorant molecules. The selected methodology elucidated about hOBP properties, including the binding modulation to pleasant and unpleasant odorants.

## 2. Methods

### 2.1. Human Odorant Binding Protein Structure

The UniProt [19] was used to obtain the full canonical amino acid sequence for hOBP, corresponding to the entry Q9NY56. Then, the hOBP full structure was predicted using the iterative threading assembly refinement (I-TASSER) server [20], a method to predict protein structure and function that uses a multiple threading approach based on templates from the Protein Data Bank (PDB) [21]. The threading templates used to generate the full hOBP structure were the proteins with the following codes: 5X7Y, 3CBC, 1EW3, 1GM6, 4RUN and 1EXS, all lipocalin-type proteins as the OBP. The I-TASSER server-generated five-hit models, and from this list, we chose the one with the best C-score (confidence).

The differences between the modeled conformation and the available hOBP X-ray structure (PDB ID: 4RUN) consist in the lack of the N-terminal in chain A, which is the chain carrying the ligand, but also the crystal structure has two different amino acids placed at the barrel core. As the binding site for hOBP is not established prior to the docking experiments or in literature, these two positions could be interacting sites with odorant molecules, hence the importance of modeling the canonical sequence. Nevertheless, the hOBP model was compared to the X-ray results, both at the conformational level and regarding docking affinities (Figures S2 and S4 and Table S3).

### 2.2. Molecular Dynamics Simulations and Choice of hOBP Conformation

Molecular dynamics (MD) simulations were performed on the hOBP complete sequence-structure model to equilibrate its conformation, although highly similar to human 4RUN X-ray conformation. The protein was inserted in a cubic box with an SPC water model, comprising a volume of 385.53 nm^3^. One stage of energy minimization was performed using a maximum of 50,000 steps and the steepest descent method. Initialization steps using canonical NVT (constant number of particles, volume and temperature) and isothermal-isobaric NPT (constant number of particles, pressure and temperature) ensembles were performed applying position restraints (with force constant of 1000 kJ·mol^−1^·nm^−2^) to all heavy atoms in both procedures. After initialization, 50 ns of production simulation took place, without restraints. All simulations were performed using the GROMACS 5.1.4 version [22,23] within the GROMOS 54a7 force field (FF) [24,25]. The Lennard-Jones interactions were truncated at 1.4 nm, and the particle-mesh Ewald (PME) [26] method for electrostatic interactions with a cutoff of 1.4 nm was used. The algorithm LINCS [27] was used to constrain the chemical bonds of the protein as well as the algorithm SETTLE [28] in the case of water.

After the simulation, RMSD and cluster analysis were used to determine the system equilibration at the most representative conformation for hOBP under solution. The single-linkage method, with a cutoff of 0.1 nm, was implemented for the last 25 ns of unrestrained simulation time, from which the RMSD is equilibrated (Figure S3). This technique clusters structures that are below the RMSD cutoff. For hOBP, only two clusters were computed, one containing 26 conformations and the second comprising 2473 structures. Within the most populated cluster, the structure which minimizes the RMSD variance among the others sampled (time 48,300 ps) was chosen to be used for docking experiments, i.e., the most representative structure of the whole simulation.

### 2.3. Odorant Molecules Setup

Quantum chemical calculations, at the DFT level, were used to prepare the odorant molecules for docking, i.e., to obtain an optimized structure for each odorant. Calculations were done with the hybrid density functional B3LYP [29] together with the 6-31 + G (d,p) basis set. All molecules were computed with the Gaussian 09 [30] suite of programs in a vacuum and without vibrational corrections. After obtaining the most stable/probable conformation, OpenBabel [31] was used to transform Gaussian outputs to the PDBQT format, suitable to be used in AutoDock Vina [32].

The physicochemical properties were collected from three preferential databases: PubChem [33], ChemSpider [34] and The Good Scents Company [35]. Whenever possible, values obtained experimentally were selected. Further, in the case of stereoisomers, preference was given to the most abundant structure in nature. The properties listed were molecular weight (MW), logP, vapor pressure (Vp; logVp was used), number of double bonds (nº DB) and the degree of unsaturation (DoU). This last property is calculated according to Equation (1), where *C* is the number of carbons, *N* the number of nitrogens, *X* is the number of halogens and *H* the number of hydrogens. For a saturated molecule (only single bonds and no ring), *DoU* will be 0.

(1) $$DoU=\frac{2C+2+N-X-H}{2}$$

### 2.4. Principal Component Analysis

Principal component analysis (PCA) from SPSS software [36] was used to correlate the data listed or obtained for the 60 odorant molecules against the binding energy predicted by using docking experiments. This methodology allows to reduce the dimensionality of data and to perceive straightforward the linear correlation among the variables. It is a way of in a single graph, to follow the relationship between the binding energy and all the properties of all odorant molecules, instead of using several linear correlation graphs (scatter plots) between only two variables (see SM), that will not reveal some pattern as in PCA.

### 2.5. Virtual Screening of Odorant Molecules

The affinity (ΔG_binding_) of our odorant library was estimated by virtual screening all the 60 molecules against the hOBP structure, using AutoDock Vina [32]. The aim was to determine if when molecules share a particular structural or physicochemical characteristic, the binding mode and energy will be similar. In addition, docking experiments allowed us to search for different interaction patterns between pleasant and unpleasant odorant molecules.

In the hOBP middle structure, the grid box was settle comprising the barrel region with 22 × 32 × 24 grid points in a grid spacing of 1 Å. An exhaustiveness of 20 and num_modes = 20 was used for each docking run. Figure S1 shows the Vina grid box settled for virtual screening, where the barrel loops and bends (extremities) were also contemplated.

## 3. Results and Discussion

The Protein Data Bank (PDB) brings together some OBP X-ray structures from vertebrates, but only one structure is available for hOBP (ID: 4RUN) [37]. In this structure, chain A (containing the ligand) lacks the N-terminal portion, which could be important for protein function, as it is placed near the bottom of the barrel, but most important two residues placed at the barrel core differ from the hOBP canonical sequence, the Ser99 and the Asn112 (see alignment in Figure S2, Supplementary Materials), and the canonical residues in place (Cys and Lys, respectively) may be interacting residues with odor molecules.

For the docking experiments, we opted to use one MD structure (not flexible docking), which was the middle structure obtained through cluster analysis. This conformation represents the most frequent arrangement of hOBP in solution, therefore indirectly reflecting a dynamic perspective of the protein. Supplementary Materials (SM) shows the RMSD curves (Figure S3) for modeled hOBP, which is very stable, considering the number of flexible turns and bends connecting the β-sheet barrel. Additionally, Figure S4 (SM) presents the superposition of our middle structure to hOBP X-ray, revealing great structural similarity and conservation.

**Figure 1:**
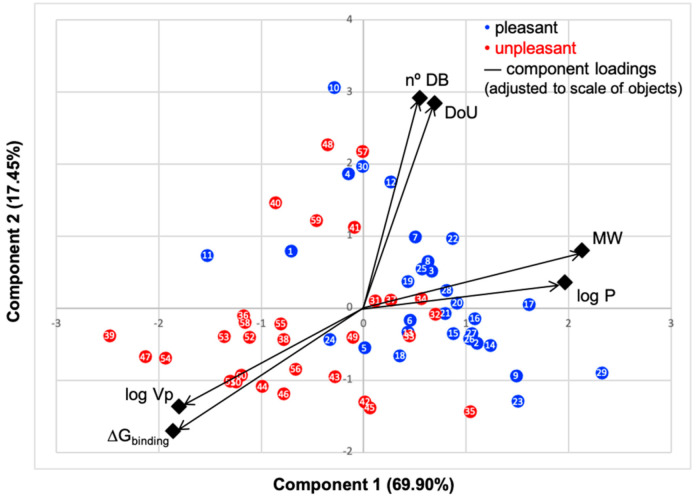
Principal component analysis (PCA) biplot of the first two components, objects factor scores and loadings, of data presented in Table 1 and Table 2. Rotated component matrix and scores were calculated using IBM SPSS Statistics software. Black color represents the loadings of the six variables scaled to objects’ values. Blue dots represent the molecules commonly perceived as pleasant odors, and red dots the unpleasant ones.

**Figure 2:**
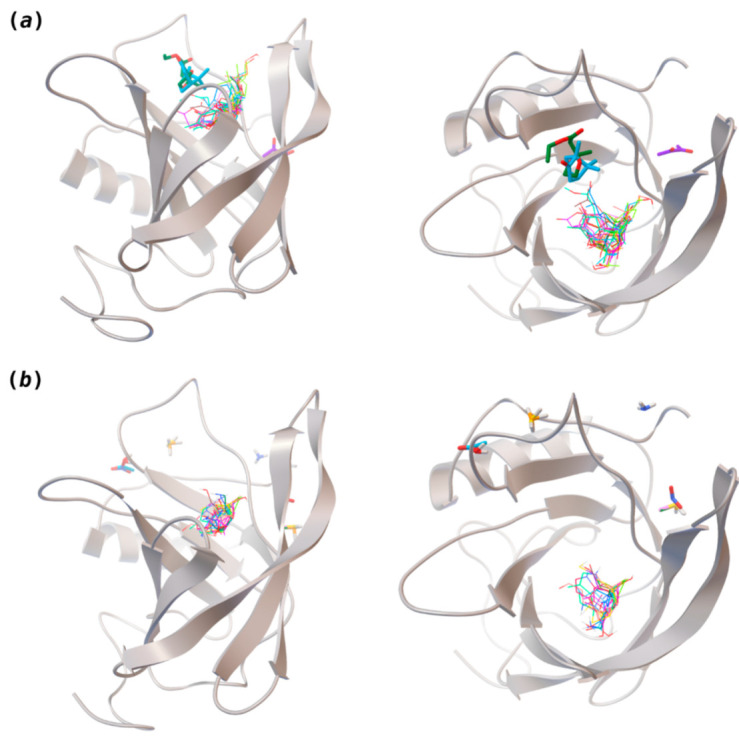
Interaction-binding modes of the selected (**a**) pleasant and (**b**) unpleasant odorant molecules, estimated with AutoDock Vina. Odorants in the most populated binding site are represented in diverse color lines, while odorants in other locations are shown in colored sticks. The human odorant-binding protein (hOBP) target is shown in the gray design.

Table 1 and Table 2, along with Figure 1 and Figure 2, present a full description of the pleasant and unpleasant odorants under study. We set a diverse range of properties to infer if odorant molecules will cluster accordingly to physical or structural properties, or, for instance, if there is a dominant property, such as hydrophobicity, volatility, etc. Considering downstream biotechnology applications of hOBP, we also find it very important to understand how the general odorant profile (pleasant versus unpleasant) correlates with the hOBP-binding properties, namely location and affinity.

The library of compounds herein was chosen to guarantee a diversified range of values in the addressed physicochemical properties. In addition, in the case of pleasant aromas, as the first studies on OBP-binding explored medium-size molecules from diverse chemical families [17,18,38], we adopted the same rationale, but taking into consideration, current odors generally used in the industry. Regarding unpleasant odors, their size range was widened, in part, due to our attempt to gather the principal body and decaying food odors or daily pungent aromas, more interesting according to our future purposes.

Looking at Table 1 and Table 2, we can infer some general differences in the properties under study between the chosen group of pleasant and unpleasant odorants; these differences between the two groups of molecules are significant according to the low *p* values and high size effects of the performed nonparametric analysis are observed (Table S2). The MW values are, on average, higher for pleasant odors. Similarly, the logP reveals more hydrophobic molecules in this category. In addition, the nº DB and the DoU values are superior for pleasant molecules, while the Vp values are overall higher for the unpleasant odorants. Regarding the calculated Gibbs free energy of binding to hOBP, the pleasant odorants presented on average more negative values than the unpleasant ones. Complementarily, binding energies were computed by using the 4RUN X-ray structure, sampling very similar results in comparison with our hOBP model (Table S3). This result is also important in order to evaluate the robustness of modeling techniques and MD simulations in predict and equilibrate protein structures, which will result in docking results in line with the screening in crystal structures.

Therefore, the combination of the differences among the properties of the chosen library of odorants leads to the hypothesis that the pleasant profile of odorant molecules may imply a high dependency on a carrier protein in order them to reach the ORs. Yet only the docking screening and the PCA analysis can unveil how the binding is structural-dependent for both classes.

PC1 represents 69.60% and PC2 17.45%; thus, over 87% of data are described by the first two components. The N°DB and DoU have some of their correlation impaired by the pleasant molecules, that is, while these properties impact the binding energy of the unpleasant molecules, for pleasant odorants, the linear correlation is very low, with an R-squared around 20% (Figures S8 and S9, SM), which results in a lower correlation in PCA plot. Although many odorants present double bonds and unsaturated moieties, these characteristics prove to be less constant through the series in their impact on the binding mode and energy. The variables logVp and ΔG_binding_ positively correlate, as they present a small angle between them, and both negatively correlate with MW and logP, due to the large angles approaching 180°. We, thus, clearly verify that the variables that most influence the binding of molecules to hOBP are logVp (volatility), logP (hydrophobicity) and MW.

Looking at the scatter plot, a distinct pattern can be perceived for the chosen pleasant and unpleasant odorants (blue and red dots), as they form two groups with minimal overlapping, indicating that in general, these aroma types behave differently. Unpleasant molecule properties correlate more with the binding energy than the pleasant ones, also seen in SM linear regressions (Supplementary Materials Figures S5–S9). This is very interesting, as it suggests that, in the selected library, the unpleasant profile preferentially modulates the binding. A less negative ΔG_binding_, indicates, in general, that the aromas are less strongly linked to hOBP. The fact that odorants from the unpleasant group present less affinity towards hOBP and that they are smaller and more hydrophilic led us to infer that, in general, they are more capable of crossing the mucus barrier by diffusion as free molecules rather than being transported by hOBP. Kinetically, this could mean a faster binding of these unpleasant odors to their specific ORs. Perhaps these unpleasant odors, connected with “hazard” or less favorable signs from the surroundings, can therefore be perceived faster than pleasant aromas. Yet, olfaction is a very complex process [39]; other factors will influence the binding, as odorant concentration, mucus viscosity and temperature, for example.

The chemical classification (see Table 1 and Table 2; 2D formula) was not tracked through PCA as a variable; nevertheless, the PCA plot can show clusters of odorants based on their similarity. For pleasant molecules, it is possible to see some aggregation consonant with the chemical function. The odorants 5, 13, 18 and 24, which are esters, appear near each other. Similarly, the terpenes 3, 7, 8, 16, 19, 20, 21, 25 and 28 are closer. Interestingly, the molecules 10 and 29, the most negatives ΔG_binding_ are farther from the other objects. Molecule 11 is an outlier in this series in terms of MW, logP and Vp, having values more similar to the ones in the unpleasant library, explaining why it is closer to the red dots.

Highlights in the unpleasant series point to molecules 39, 47 and 54, shifted from others, which are some of the molecules outside the barrel. In fact, these odorants have the highest Vp of the series, making them outliers from the series. The molecules 31, 32, 33, 34 and 37, all with MW above 120.00 g/mol, are the molecules that are mixed with the pleasant ones. Importantly, the molecules 48 and 57, which clusters together, have the higher DoU values and are the ones with most negative ΔG_binding_, this is probably due to this characteristic jointly with the MW value. The compound classification, however, is not a strong differentiating factor for the binding location, as the majority of compounds bind to the same hOBP place (Figure 2).

The binding of pleasant odorants occurs mostly at the same location (Figure 2a), at the top of the barrel. The few exceptions are observed for camphor (6), and fructone (13), which bind a little shifted from the rest of the series, and for diacetyl (11), which binds out of the barrel core. Diacetyl is the lightest pleasant molecule and also the most hydrophilic and volatile, as indicated by the values of MW, logP and Vp, which may explain the differentiated binding to hOBP. Regarding camphor, fructone and diacetyl, their most negative binding energies are associated with a binding site different from the other pleasant odorants; however, their following docking positions resembled the preferable binding location as for the other pleasant molecules.

Looking at the unpleasant odors (Figure 2b), the location of the binding sites is much more variable among molecules. The two acids, acetic acid (36) and nitric acid (52), bind at a similar position and are external to the barrel, with a very similar ΔG_binding_. Ammonia (39) is also outside the barrel, but in a different location than the acids, as well as the pair phosphine (54) and hydrogen sulfide (47), and the trio methanethiol (50), methyl phosphine (51) and nitrogen dioxide (53). For the latter, the binding energies vary from −0.6 up to −2.8 kcal, which may suggest that these molecules may not even bind to OBP at all. Remarkably, the Vp of these molecules is the biggest on the unpleasant list, being the most volatile odorants. In addition, they are small and hydrophilic molecules with lower logP, which may facilitate the direct access to olfactory receptors by diffusion in the mucus, without the participation of OBP as a carrier.

The majority of the unpleasant molecules bind to the same site as the pleasant ones, making it possible to establish a binding site for hOBP. Yet, it is important to highlight that the binding energy varies considerably between these groups as a consequence of the intrinsic properties of the odorants. In fact, in 2002, Briand and coworkers [40] suggested that hOBP can discriminate among odors. They stated that OBP binds fatty acids rather than aldehydes and larger aldehydes, preferably to other chemical functions, concluding that hOBP binds more efficiently longer chains. Throughout our odors’ list, longer and bulky molecules as hedione (17), mefrosol (22) and sandalore (29), with 13, 12 and 14 carbons, respectively, present strong affinity towards hOBP. Remarkably, in the unpleasant group, the aldehyde 2-nonenal (32), which has one of the most negative ΔG_binding_, has the longest hydrocarbon chain from the series.

In 2015, Di Pietrantonio and colleagues [41] developed a bio-electronic nose based on OBPs from pig and bovine, which was able to distinguish between octenol (mushroom, human breath and sweat) and carvone (mint), revealing a different sensitivity to pleasant and unpleasant molecules, in line with our findings.

If we compare the binding of 1-aminoanthracene (1-AMA) to porcine OBP (pOBP) [11] with the bindings calculated here for hOBP, in the former, 1-AMA binds almost on the edge of the barrel, while in hOBP, the binding is slightly more internal. Although it can be mentioned that hOBP receives the typical binding of hydrophobic molecules to lipocalins barrels and, structurally, OBPs from vertebrates are very similar, the amino acid sequence differs considerably, which may affect the hydrophobic interactions between the pair ligand–receptor. Hence it is of most importance the understanding of the binding site and energy in hOBP, as it is a less studied target to date. The amino acids frequently participating in the binding are Val49, Phe 66, Phe68, Ile79, Ile99 and Lys97. Both Phe residues are able to interact via π-stacking in a few cases.

**Figure 3:**
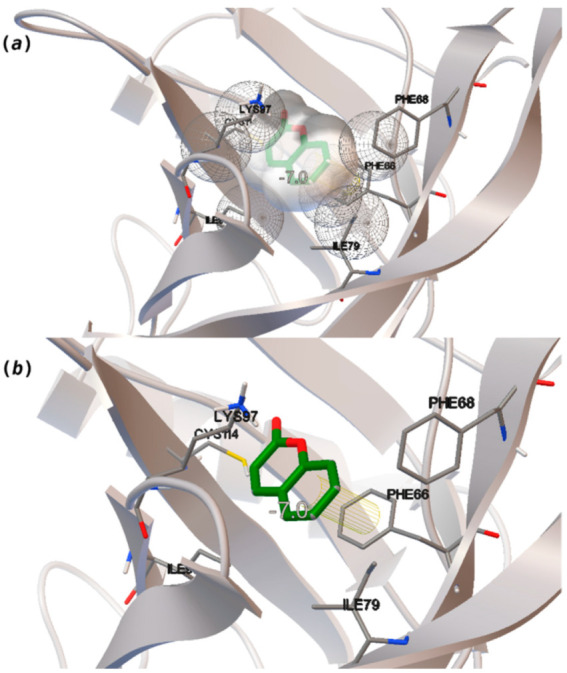
Interaction binding modes of coumarin (10) in hOBP, estimated with AutoDock Vina. In (**a**), van der Waals/hydrophobic interactions are represented, and in (**b**), a pi-pi interaction between coumarin ring and Phe residue. Coumarin is shown in green sticks and amino acids participating in the binding, from hOBP, in gray lines.

Coumarin (10) stands out from the other pleasant odorants with the higher ΔG_binding_. The affinity to hOBP ranges from −3.6 kcal/mol to −7.0 kcal/mol within the selected group of pleasant odors, mostly resulting from a combination of the physicochemical properties MW, logP and Vp, where one or two of these variables have a greater role in binding. The MW and the logP are reflected in the number of hydrophobic atoms that interact with the hOBP, contributing in this way to the sum of forces in the docking algorithm, especially in van der Waals interactions. Yet, in the particular case of coumarin, a π-stacking interaction must have a higher weight leading to the highest binding affinity (Figure 3), even though coumarin does not have the highest MW or logP.

## 4. Conclusions

OBP is well established as a key player in the sense of smell, being responsible for carrying and delivering the odorant molecules to the ORs [42]. In the present study, we address the binding of 60 odorant molecules to a very robust model of hOBP complete sequence, based on the 4RUN X-ray structure. Our findings demonstrate that the MW, the logP (hydrophobicity level), and the vapor pressure (Vp–volatility) are the physicochemical properties that impact more the ΔG_binding_, being the chemical classification, the number of double bonds and the degree of unsaturation, less crucial variables for the binding event. Our findings suggested that for the selected unpleasant odorants, the above-cited properties correlate more with ΔG_binding_ than for the pleasant molecules. According to the here reported *in silico* data, OBP discriminates between molecules from the pleasant and unpleasant groups of chosen odorants, not only by the binding site but also and mainly through the “binding strength”. From a biotechnological perspective, the fact that OBP will bind preferentially “pleasant” odors than “unpleasant” ones or that pleasant odors may displace unpleasant ones from OBP is particularly important and useful for future OBP applications, such as sensors systems for the assessment of food contamination or for the evaluation of indoor air quality in buildings, as suggested by Di Pietrantonio [41].

Odor scientists, as well as fragrance professionals, have tried to establish comprehensive standards for the description, measurement, and prediction of odor quality characteristics. An olfactory classification system to define a perceptual space and facilitate objective communication about odors has not yet been found. Not all chemical molecules entering the nasal mucosa produce odor sensations, but those that do produce will vary in profile—pleasant or unpleasant—and intensity. Pleasantness is a significant aspect of odor perception: the neuronal processing of odors and emotions are partly overlapping in limbic structures, and the close connection is rooted in a point of human evolution when odors informed us on what to approach and what to avoid [43]. The odor quality and character also depend on odorant concentration, which was not contemplated in this work. The complexity is raised if the odor is a mixture of odorant molecules. In this case, the odor quality and character also depend on the organoleptic purity and not so much on the chemical purity. Looking just at the physicochemical properties here analyzed will hardly be sufficient to predict if a certain molecule can be perceived as pleasant or unpleasant. In spite of that, the findings of this work, in such a small group of odorant molecules compared to the odor space, are very relevant and may provide important clues for researchers devoted to developing predictive models of odor quality.

In this study, we found a clearly different pattern of hOBP-binding among the selected groups of pleasant and unpleasant odors, based on the MW, hydrophobicity and volatility of odorants. Given that, this work is important in emphasizing the role of OBP in human olfaction and in drawing attention to the possible role of this group of auxiliary proteins in the olfactory code.

This work is a first step in understanding the relationship between the odorant profile and its connection to hOBP, which can help in the technological application of aromas or in the development of sensors that mimic the function of this protein. Nevertheless, future steps in this research will go through the design of one or more OR structural models to assess the delivery of odorants to these important receptors and how OBP interacts with both classes of ORs.

## Figures and Tables

**Table 1:** Description of pleasant odorant molecules under study, according to the physicochemical and structural characteristics.

	Formula	Name and Odor Description	MW (g/mol)	log P	Vp (mmHg)	nº DB	DoU	ΔG_binding_ (kcal/mol)
1	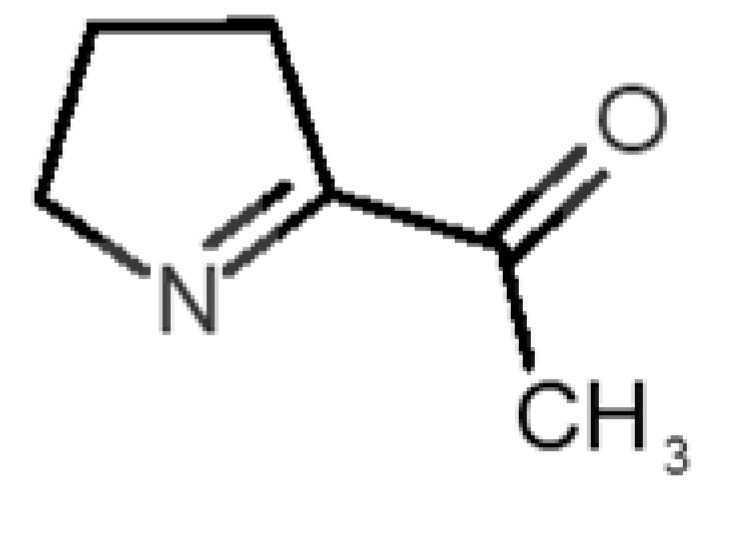	*2-acetyl-1-pyrroline* (roasted/bread)	111.14	−0.02	0.793	2	3	−4.5
2	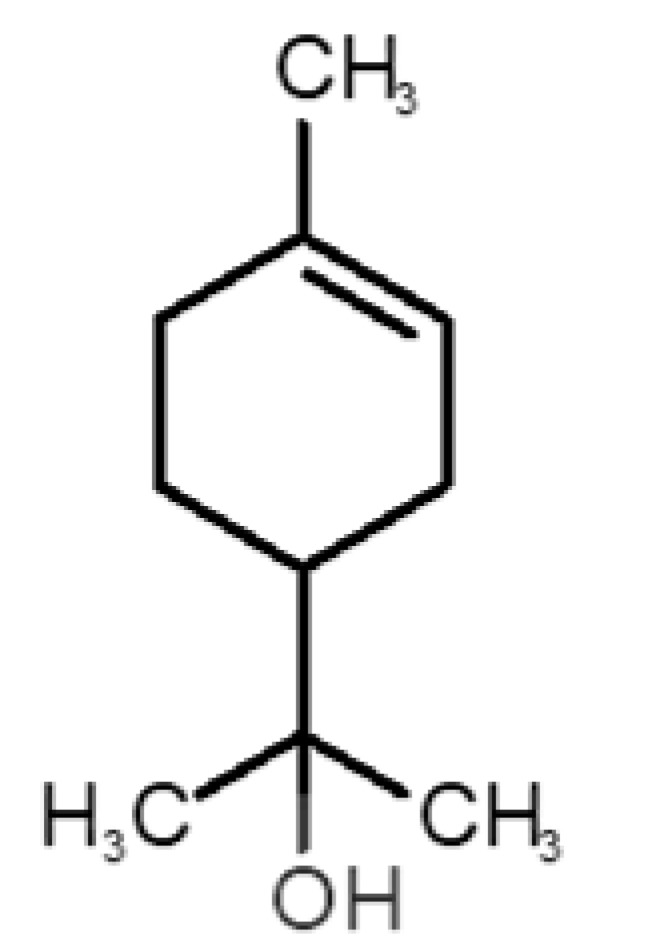	*α-terpineol* (lilac)	154.25	2.98	0.042	1	2	−5.9
3	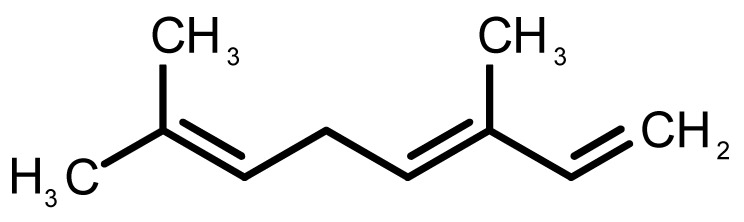	*β-ocimene* (sweet herbal)	136.24	4.41	1.559	3	3	−5.9
4	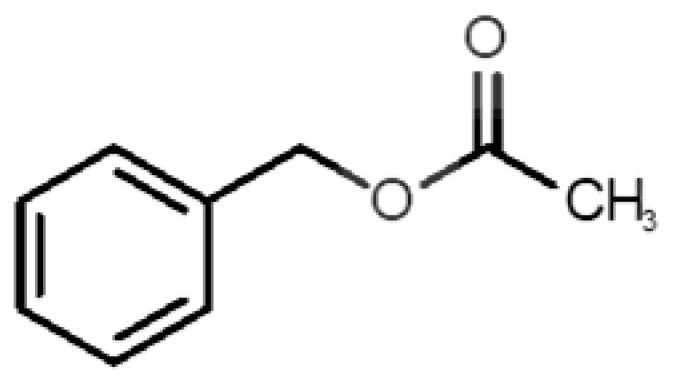	*benzyl acetate* (strawberry/pear)	150.17	1.96	0.177	4	5	−5.8
5	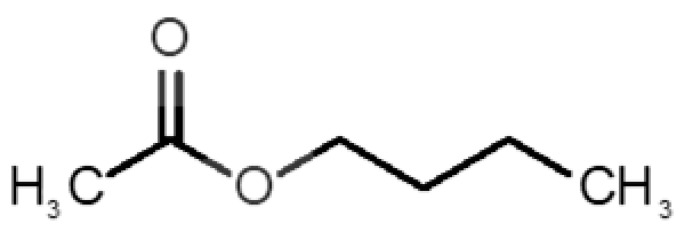	*butyl acetate* (banana)	116.16	1.78	11.500	1	1	−4.1
6	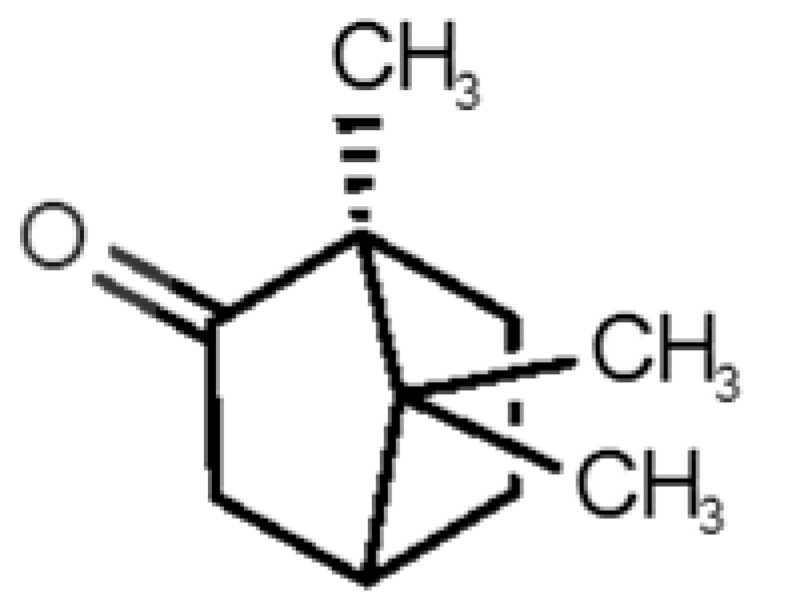	*camphor* (camphor)	152.24	2.38	0.650	1	3	−4.3
7	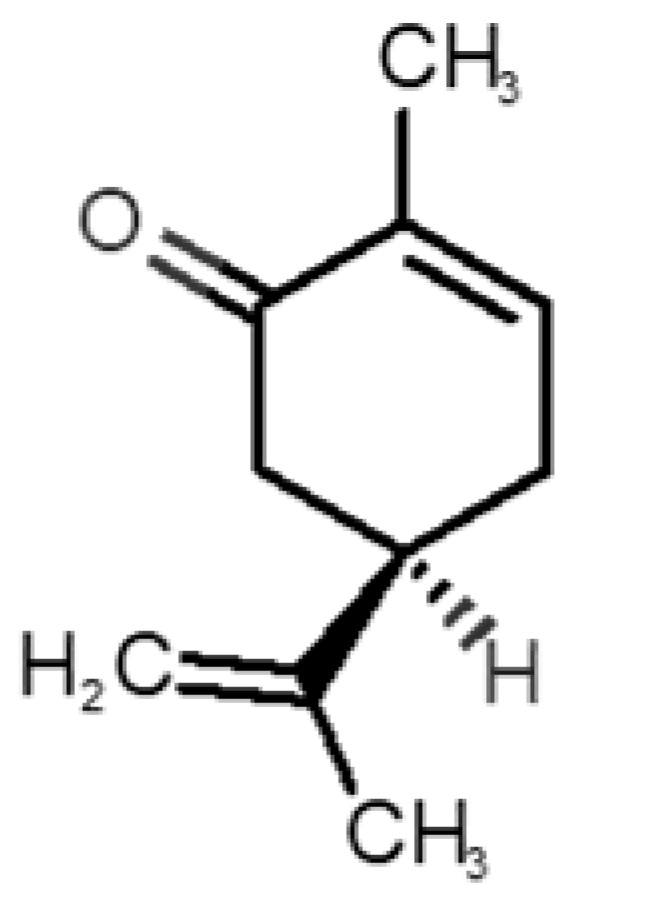	*carvone* (mint)	150.22	3.07	0.115	3	4	−6.2
8	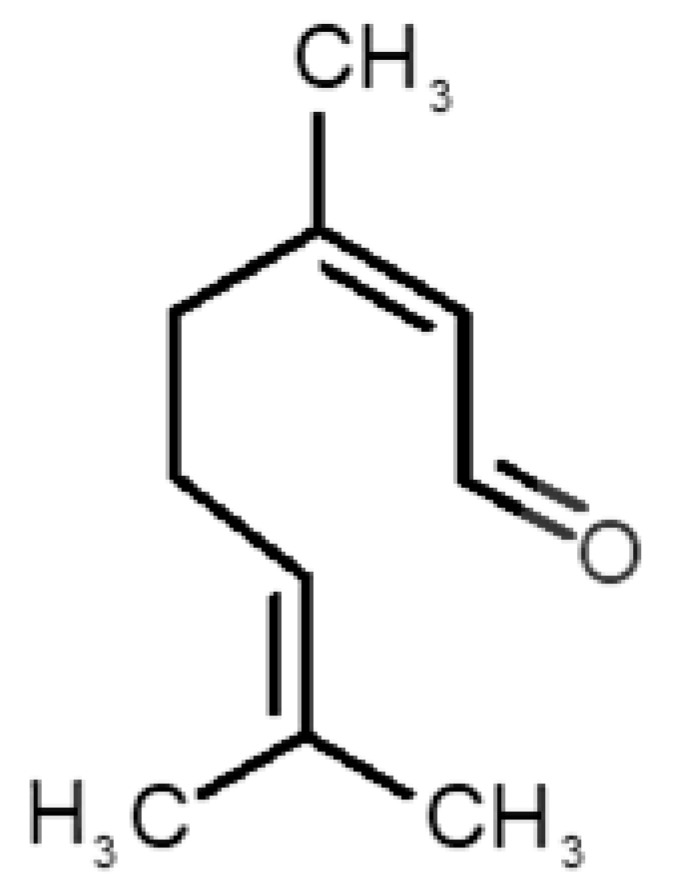	*citral* (lemon/citrus)	152.24	3.17	0.091	3	3	−5.9
9	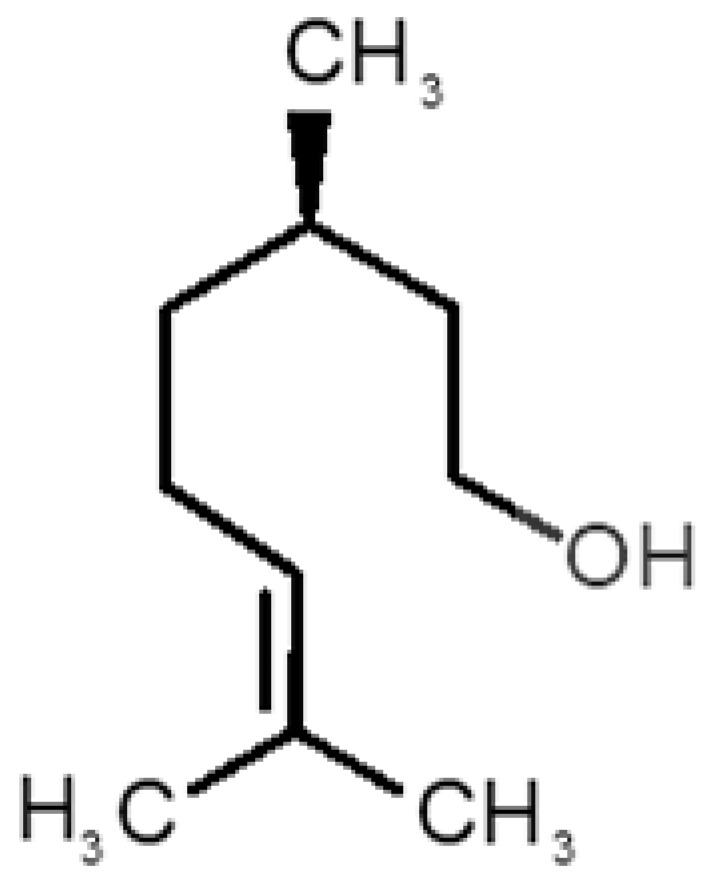	*citronellol* (citronella/rose-like)	156.27	3.91	0.020	1	1	−5.7
10	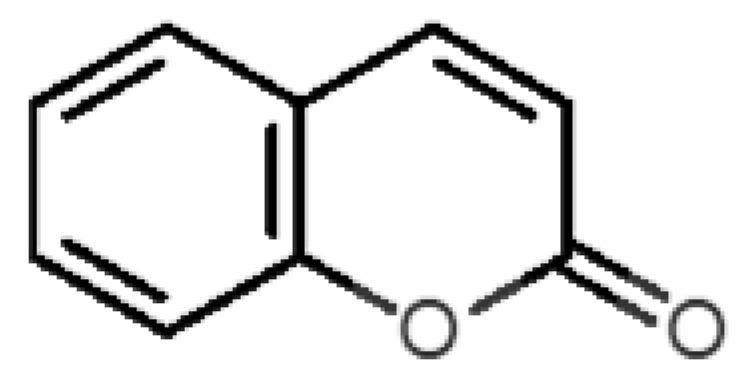	*coumarin* (sweet vanilla)	146.15	1.39	0.001	5	7	−7.0
11	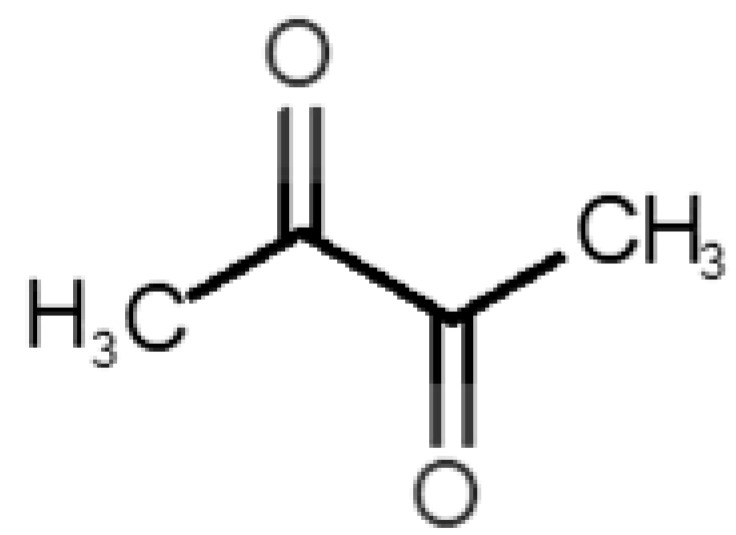	*diacetyl* (buttery)	86.09	−1.34	56.800	2	2	−3.6
12	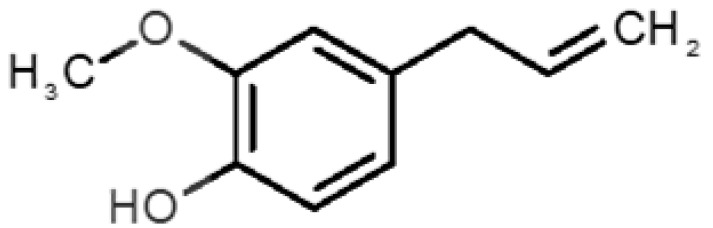	*eugenol* (cloves)	164.20	2.49	0.022	4	5	−6.1
13	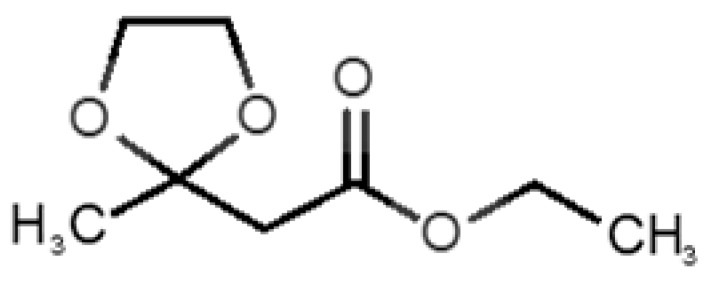	*fructone* (apple)	174.19	0.98	0.219	1	2	−4.2
14	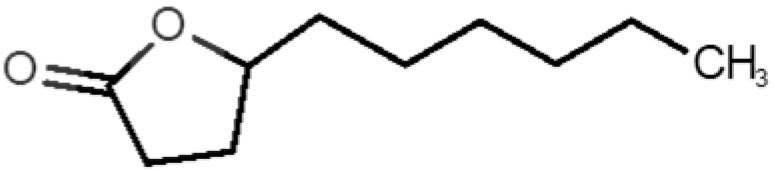	*gamma decalactone* (coconut)	170.25	2.72	0.005	1	2	−5.5
15	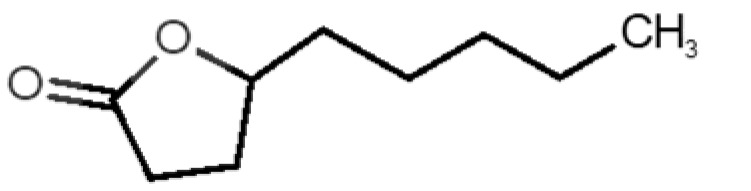	*gamma nonalactone* (peach/fruity)	156.23	1.94	0.009	1	2	−5.5
16	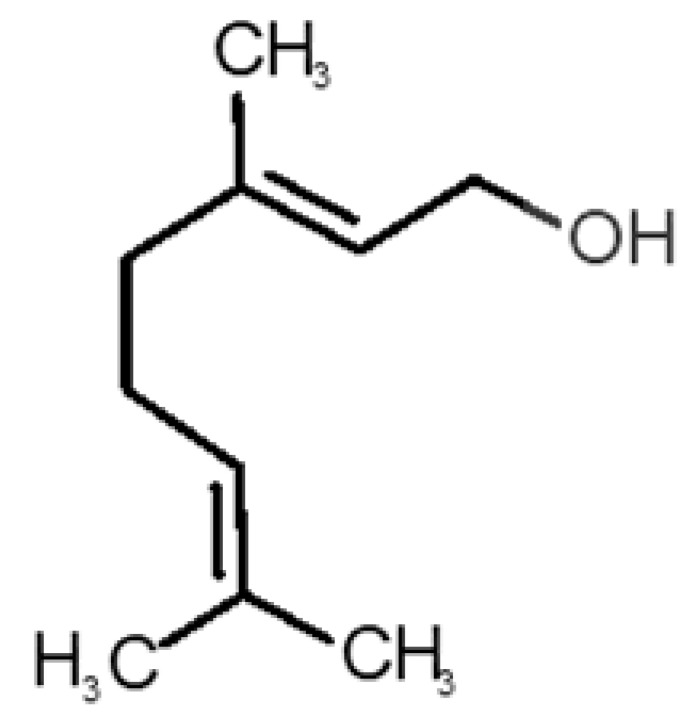	*geraniol* (floral/sweet rose)	154.24	3.56	0.030	2	2	−5.8
17	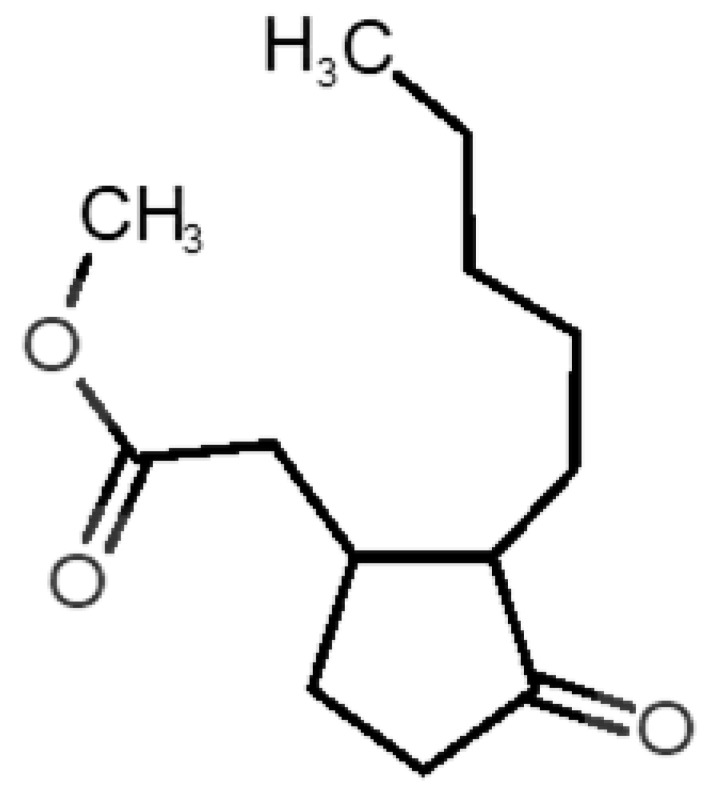	*hedione* (floral/jasmine)	226.32	2.65	0.001	2	3	−6.2
18	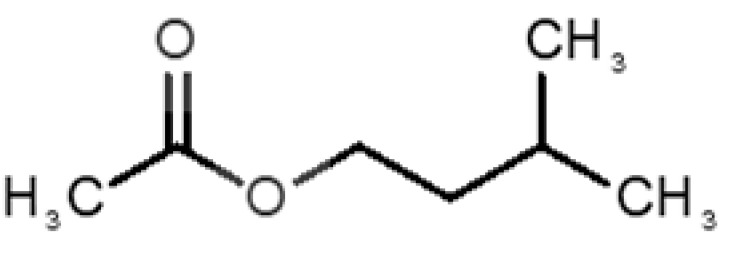	*isoamyl acetate* (pear/banana)	130.19	2.26	5.600	1	1	−4.5
19	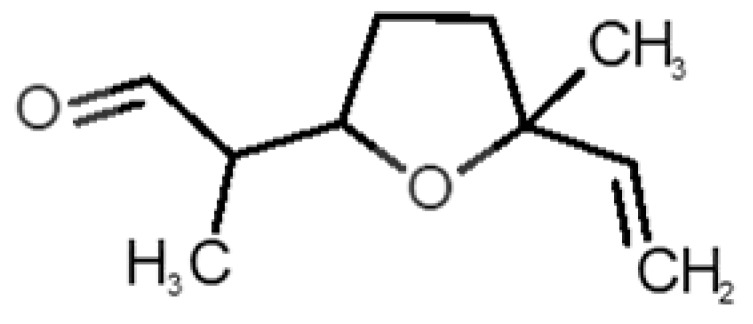	*lilac aldehyde* (floral/lilac)	168.24	1.59	0.100	2	3	−5.2
20	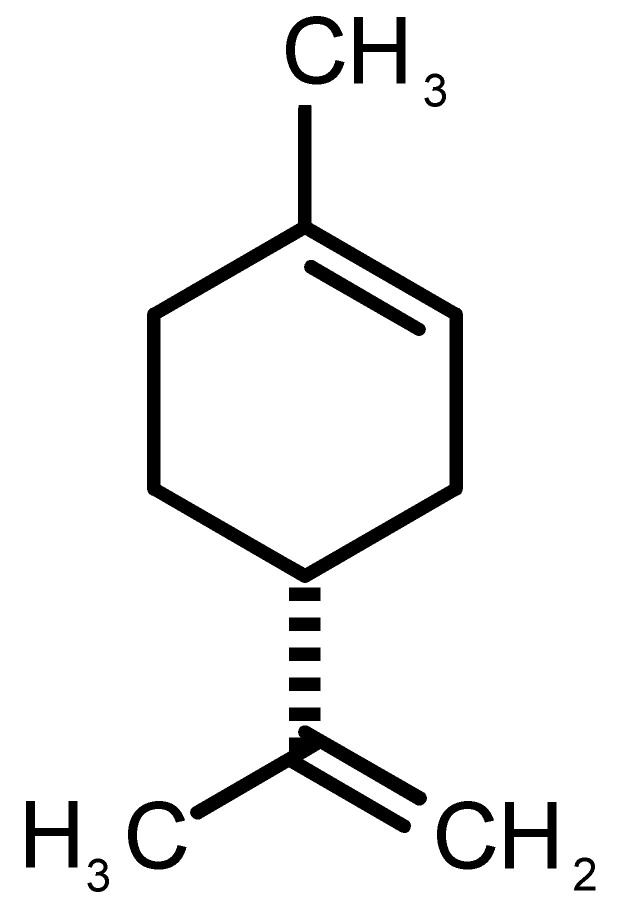	*limonene* (citric)	136.23	4.57	1.550	2	3	−6.1
21	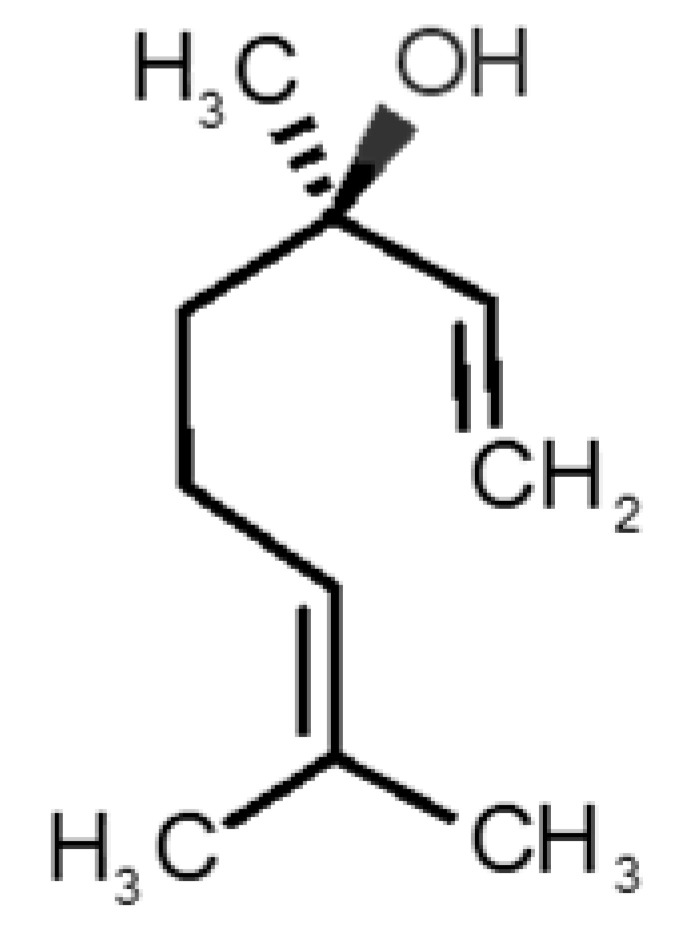	*linalool* (lavender/bergamot)	154.25	2.97	0.160	2	2	−5.5
22	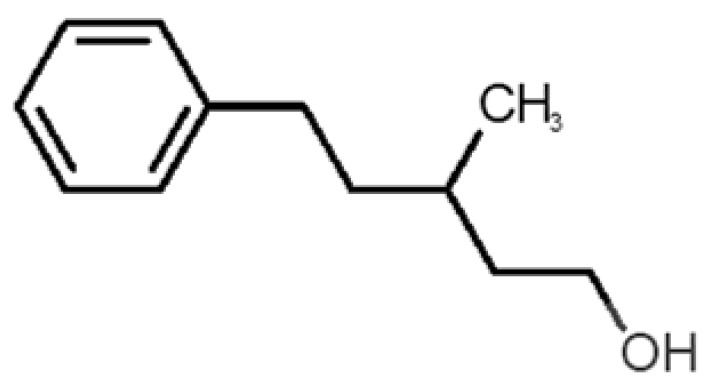	*mefrosol* (floral/rose)	178.27	2.70	0.006	3	4	−6.6
23	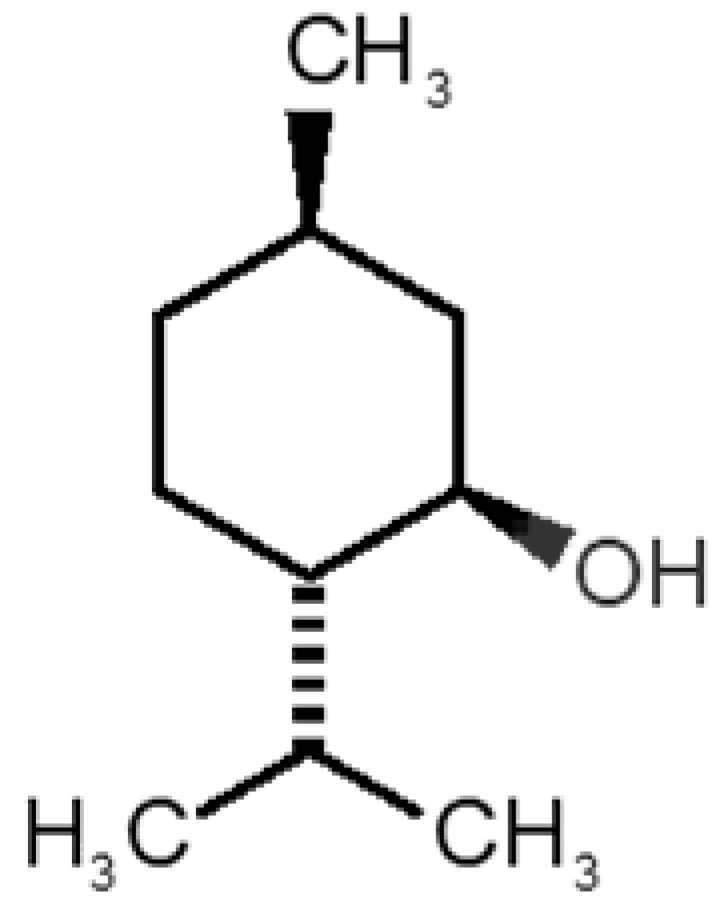	*menthol* (peppermint)	156.26	3.40	0.032	0	1	−5.7
24	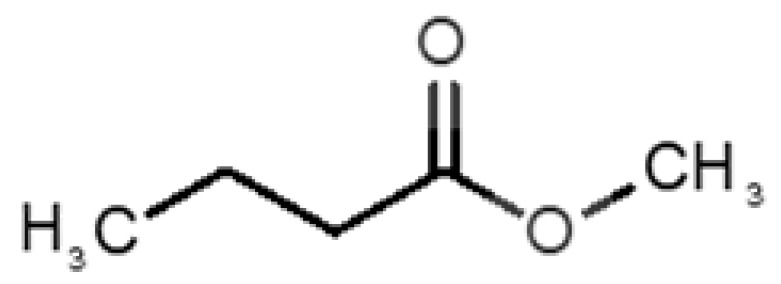	*methyl butyrate* (apple/pineapple)	102.13	1.29	32.300	1	1	−3.9
25	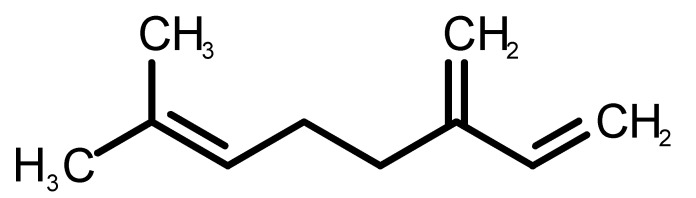	*myrcene* (herbal/woody)	136.24	4.17	2.290	3	3	−5.8
26	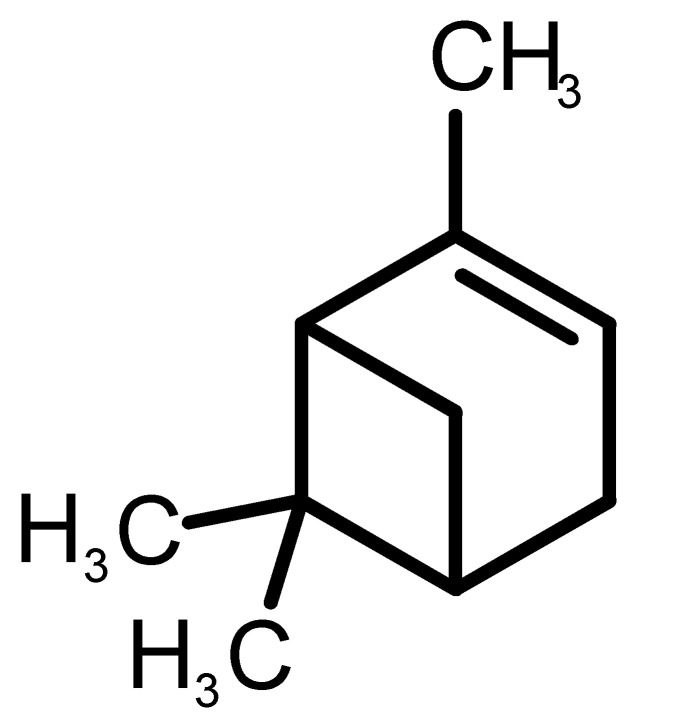	*pinene* (pine)	136.24	4.83	4.750	1	3	−5.6
27	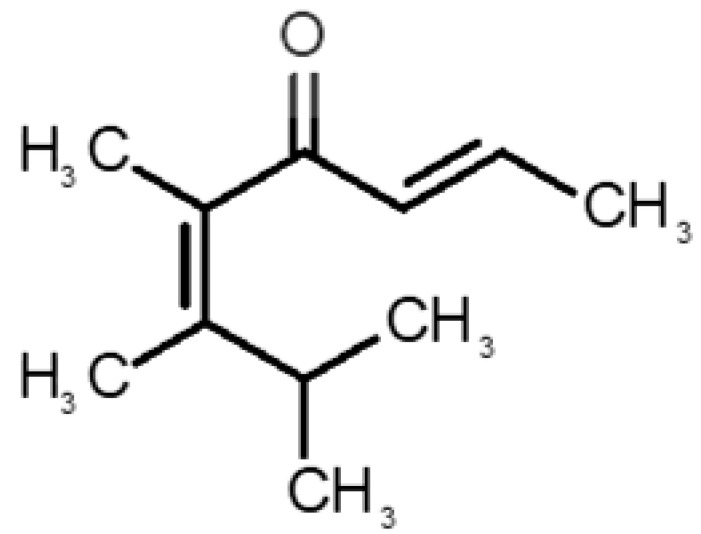	*pomarose* (plums/apples rose)	166.26	2.68	0.048	2	1	−5.9
28	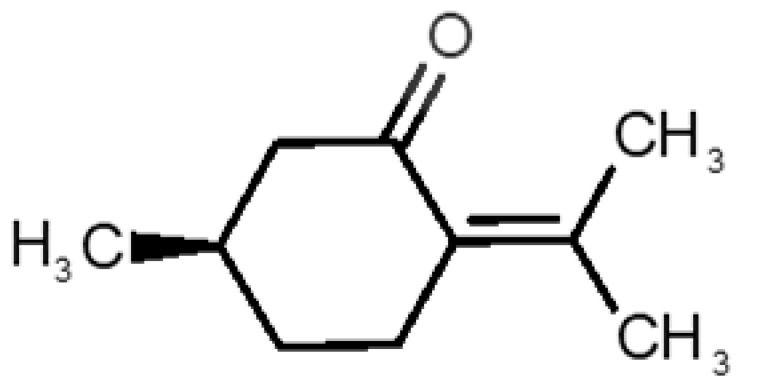	*pulegone* (peppermint)	152.24	3.08	0.123	2	3	−6.2
29	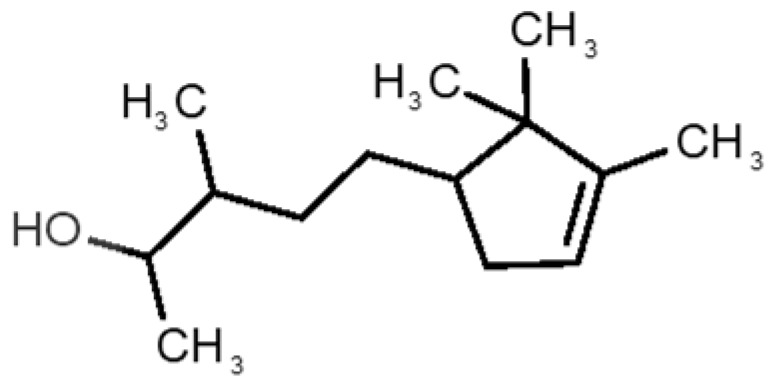	*sandalore* (sandalwood)	210.36	4.58	0.001	1	2	−6.6
30	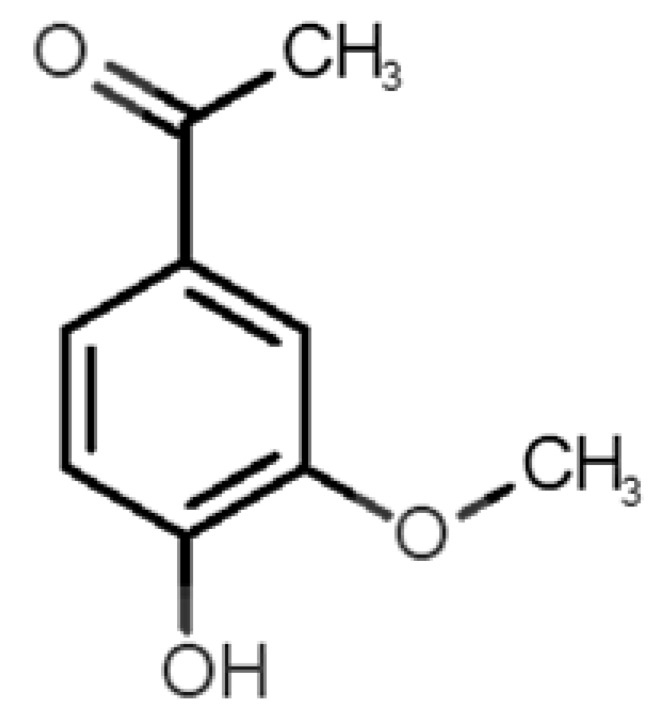	*vanillin* (vanilla)	152.15	1.21	0.000	4	5	−5.3

**Table 2:** Description of unpleasant odorant molecules under study, according to physicochemical and structural characteristics.

	Formula	Name and Odor Description	MW (g/mol)	log P	Vp (mmHg)	nº DB	DoU	ΔG_binding_ (kcal/mol)
31	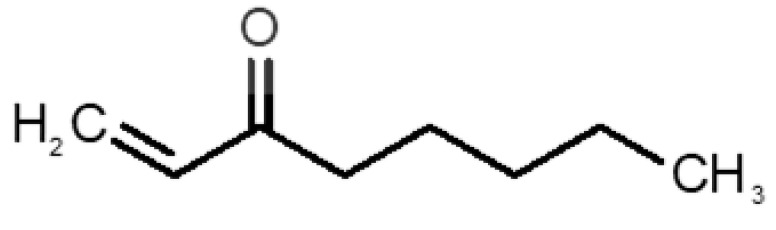	*1-octen-3-one* (metallic mushroom/blood)	126.20	2.18	1.063	2	2	−4.6
32	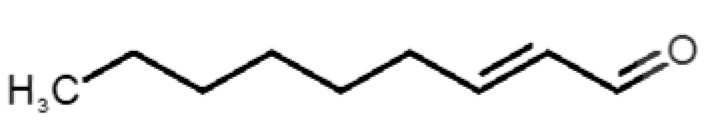	*2-nonenal* (aging body/fatty)	140.22	3.32	0.256	2	2	−5.2
33	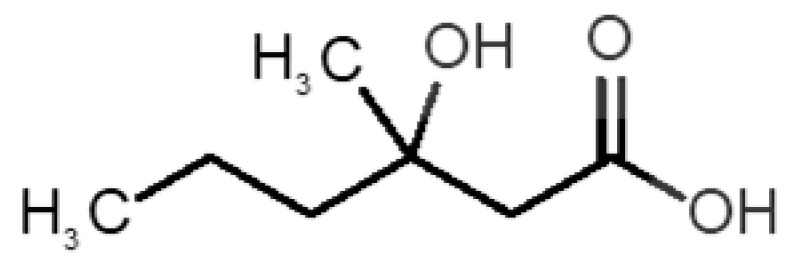	*3-hydroxy-3-methylhexanoic acid* (axillary sweat/cumin)	146.18	0.27	0.001	1	1	−4.7
34	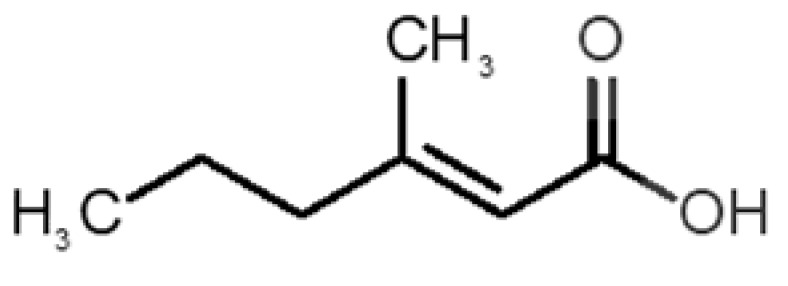	*3-methyl-2-hexenoic acid* (axillary sweathircine (goat))	128.17	2.20	0.001	2	2	−5.0
35	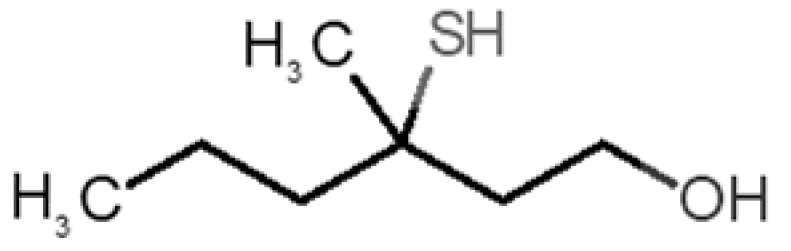	*3-methyl-3-sulfanyl-hexanol* (axillary sweat/onion)	148.27	2.15	0.023	0	0	−4.3
36	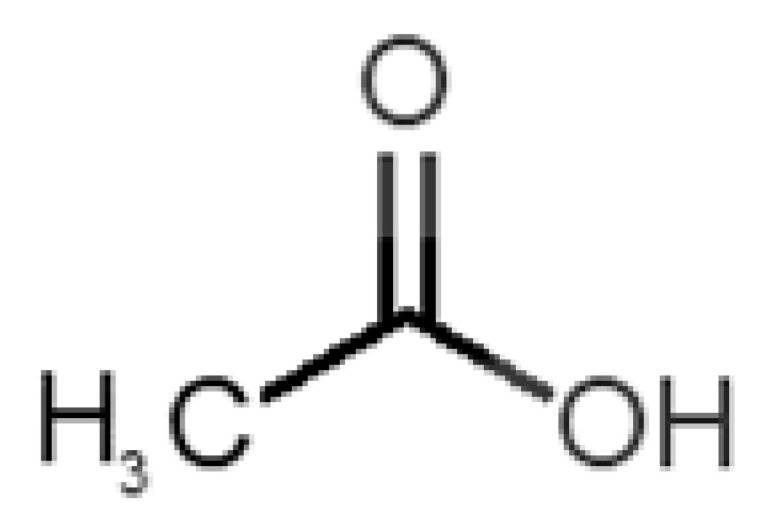	*acetic acid* (vinegar-like/pungent)	60.05	−0.17	15.730	1	1	−3.0
37	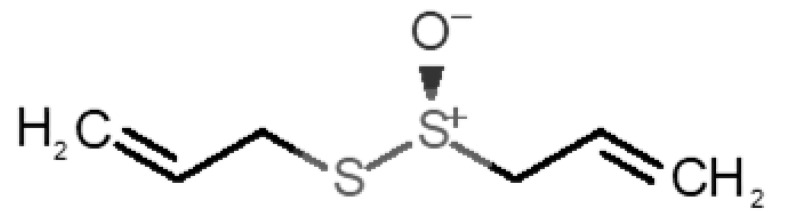	*allicin* (garlic-like)	162.27	1.13	0.038	2	2	−4.1
38	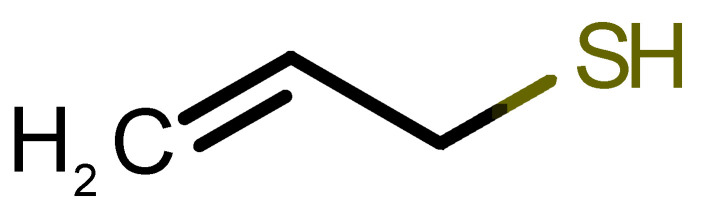	*allylthiol* (garlic/sulfurous)	74.14	1.51	151.700	1	1	−2.6
39	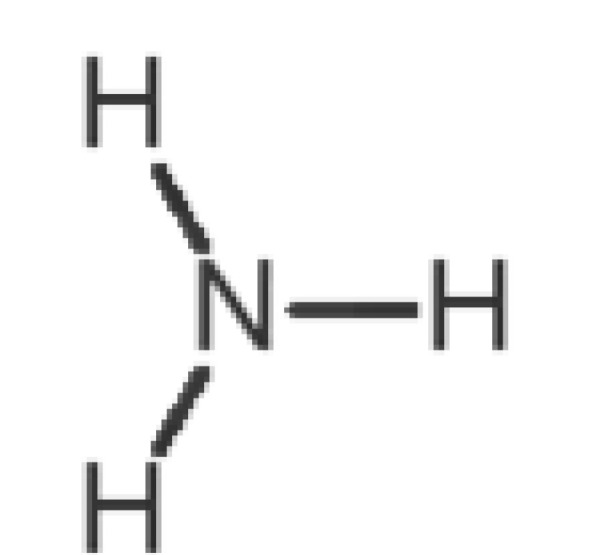	*ammonia* (pungent/sharp)	17.03	−2.66	7500	0	0	−1.4
40	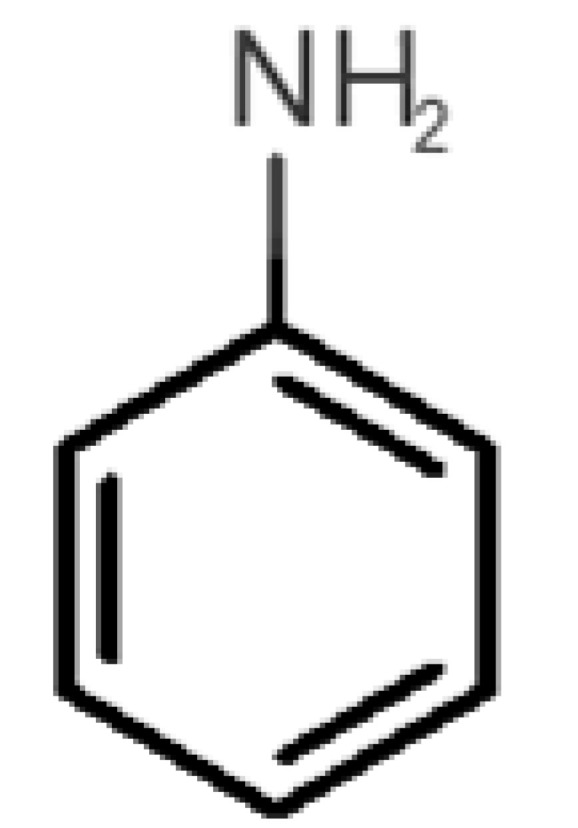	*aniline* (rotten fish)	93.13	0.9	0.667	3	4	−4.8
41	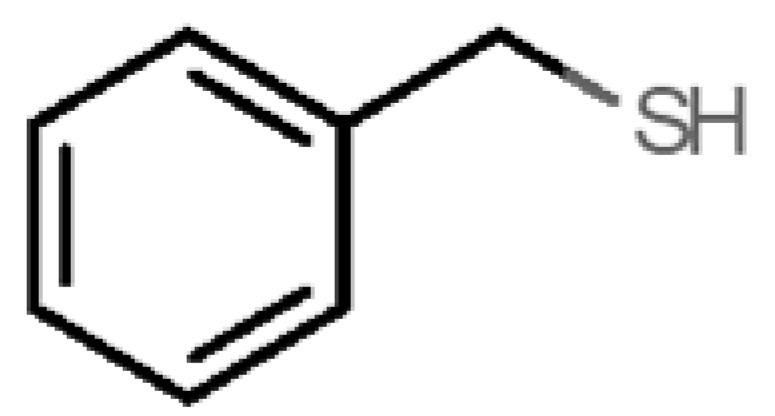	*benzyl mercaptan* (unpleasant, strong)	124.20	2.5	0.470	3	4	−5.2
42	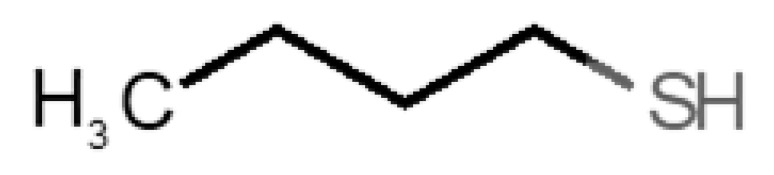	*butyl mercaptan* (skunk)	90.18	2.28	45.500	0	0	−3.3
43	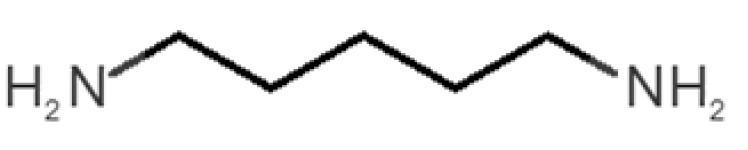	*cadaverine* (semen/vagina infection)	102.18	−0.16	1.010	0	0	−3.6
44	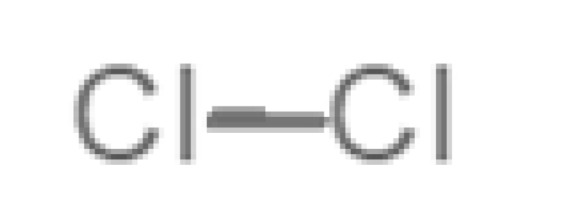	*chlorine* (pungent/irritating)	70.90	0.85	5830	0	0	−1.9
45	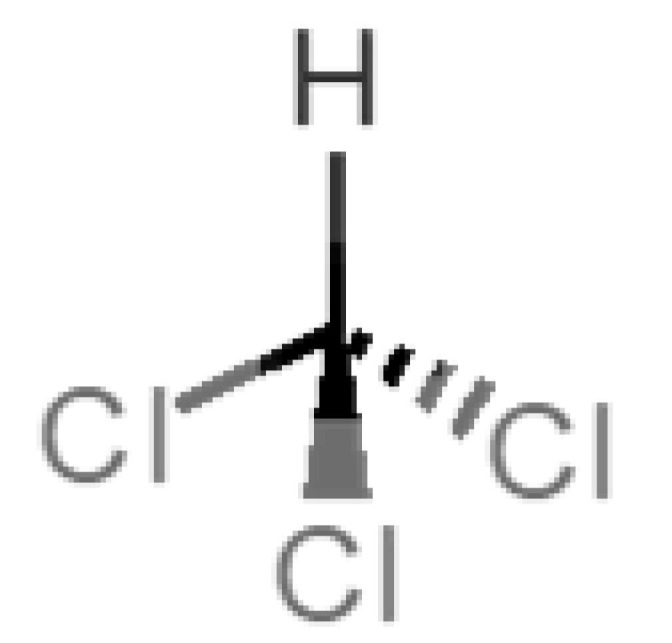	*chloroform* (ether-like/pungent)	119.37	1.97	197	0	0	−2.9
46	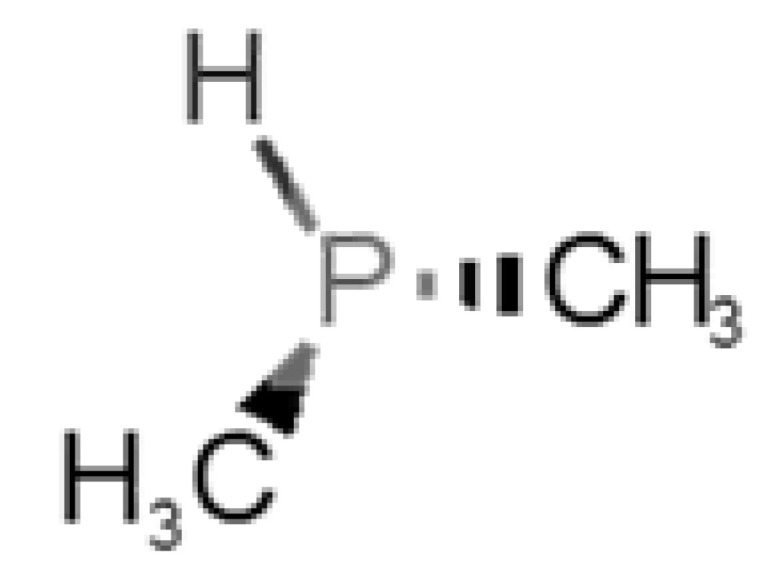	*dimethylphosphine* (metallic/fish/garlic)	62.05	1.67	760	0	0	−1.6
47	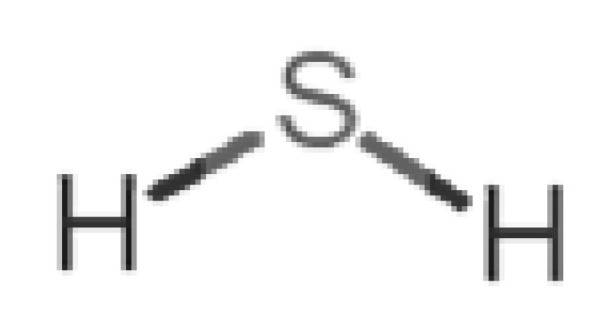	*hydrogen sulfide* (rotten eggs)	34.08	−1.38	13,376	0	0	−0.6
48	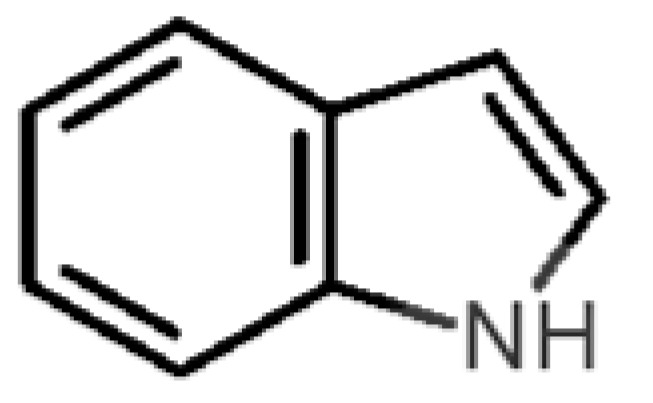	*indole* (feces)	117.15	2.14	0.0122	4	6	−5.9
49	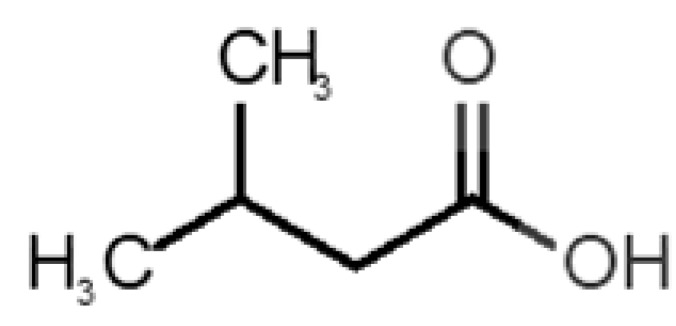	*isovaleric acid* (cheesy/sweaty/foot)	102.13	1.16	0.440	1	1	−4.1
50	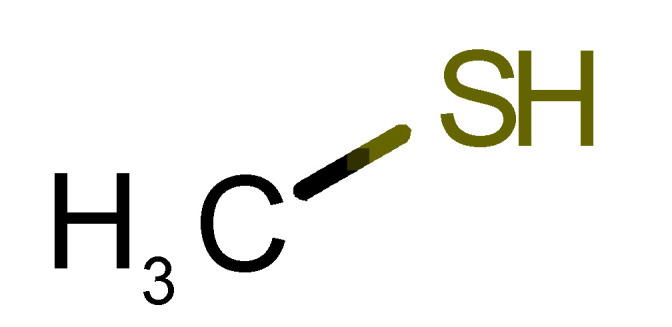	*methanethiol* (rotten cabbage/flatulence)	48.11	0.78	1510	0	0	−1.1
51	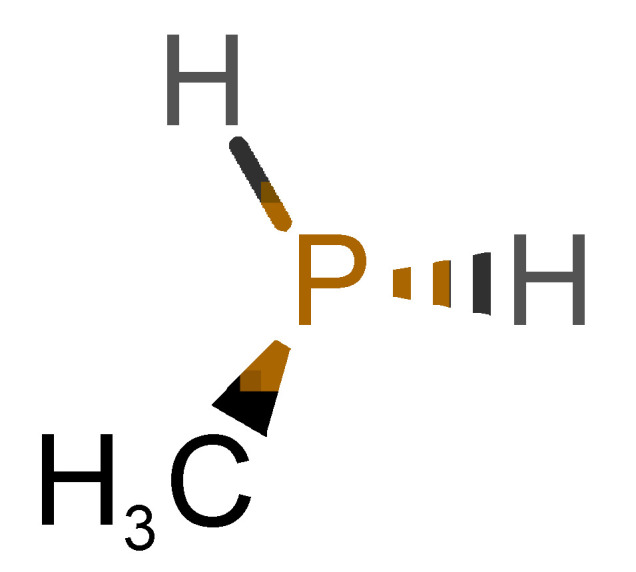	*methylphosphine* (fish-like/garlic-like)	48.02	0.70	2812.800	0	0	−1.1
52	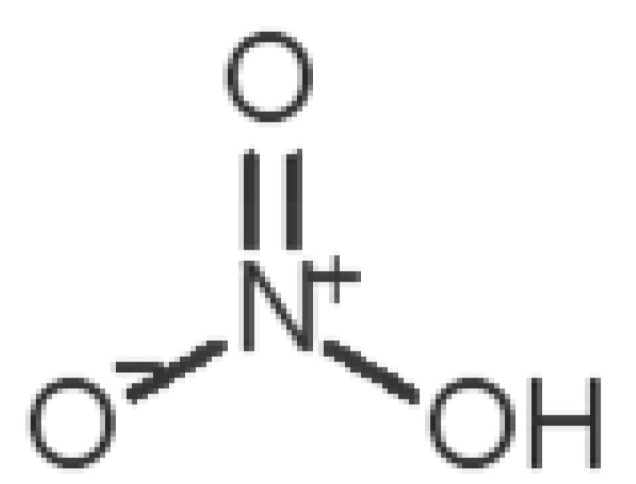	*nitric acid* (acrid/suffocating)	63.013	−0.21	63.100	1	0	−3.1
53	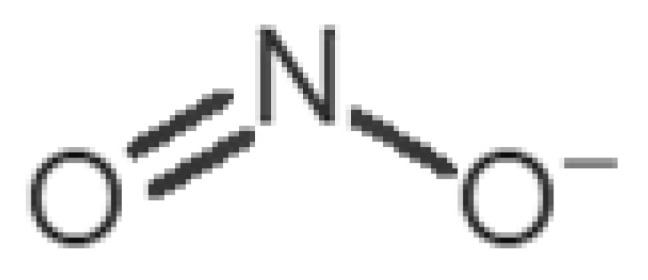	*nitrogen dioxide* (pungent/acrid)	46.006	0.06	720	1	0.5	−2.8
54	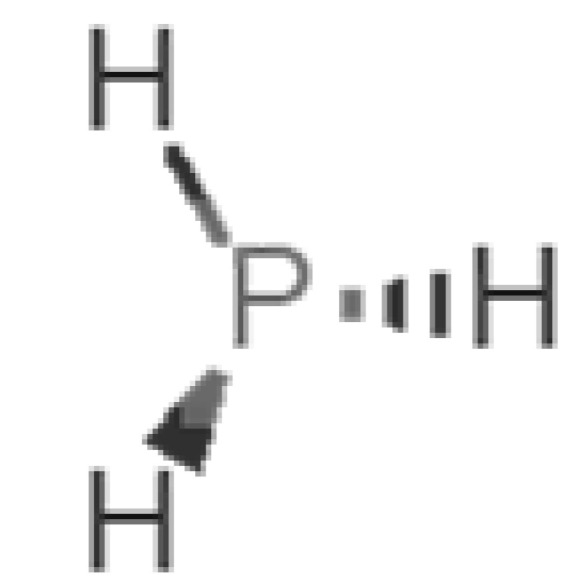	*phosphine* (rotten fish/garlic)	33.99	−0.27	29,300	0	0	−0.6
55	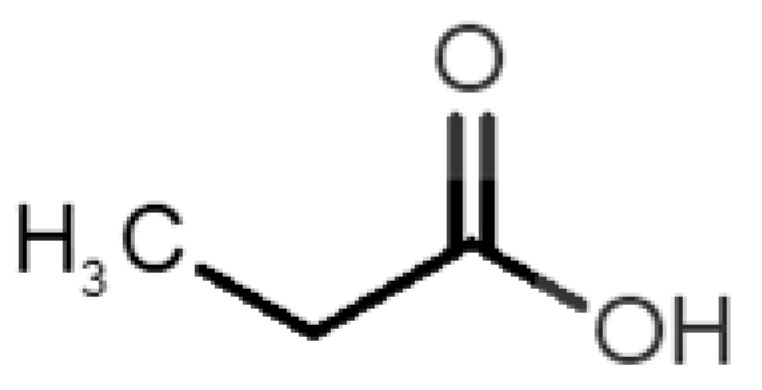	*propanoic acid* (foot/sharp rancid)	74.08	0.33	3.530	1	1	−3.2
56	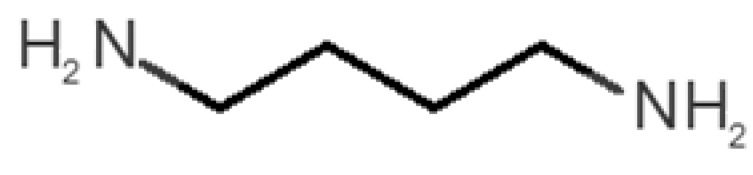	*putrescine* (putrefying flesh)	88.15	−0.7	2.330	0	0	−3.1
57	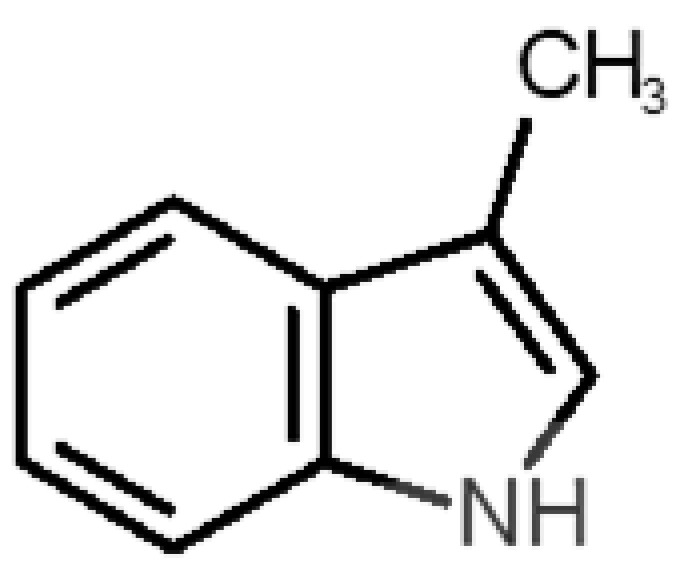	*skatole* (feces)	131.17	2.6	0.010	4	6	−6.5
58	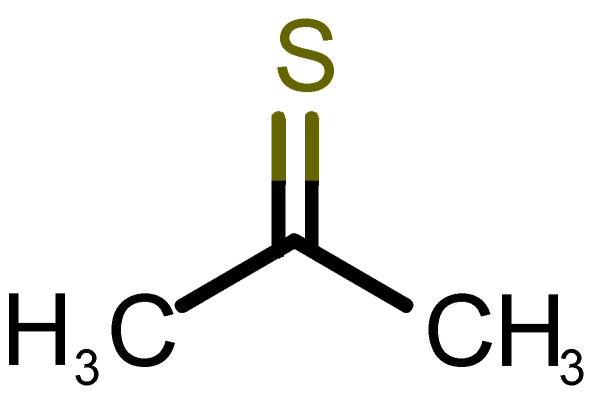	*thioacetone* (putrid)	74.15	0.05	211.500	1	1	−2.8
59	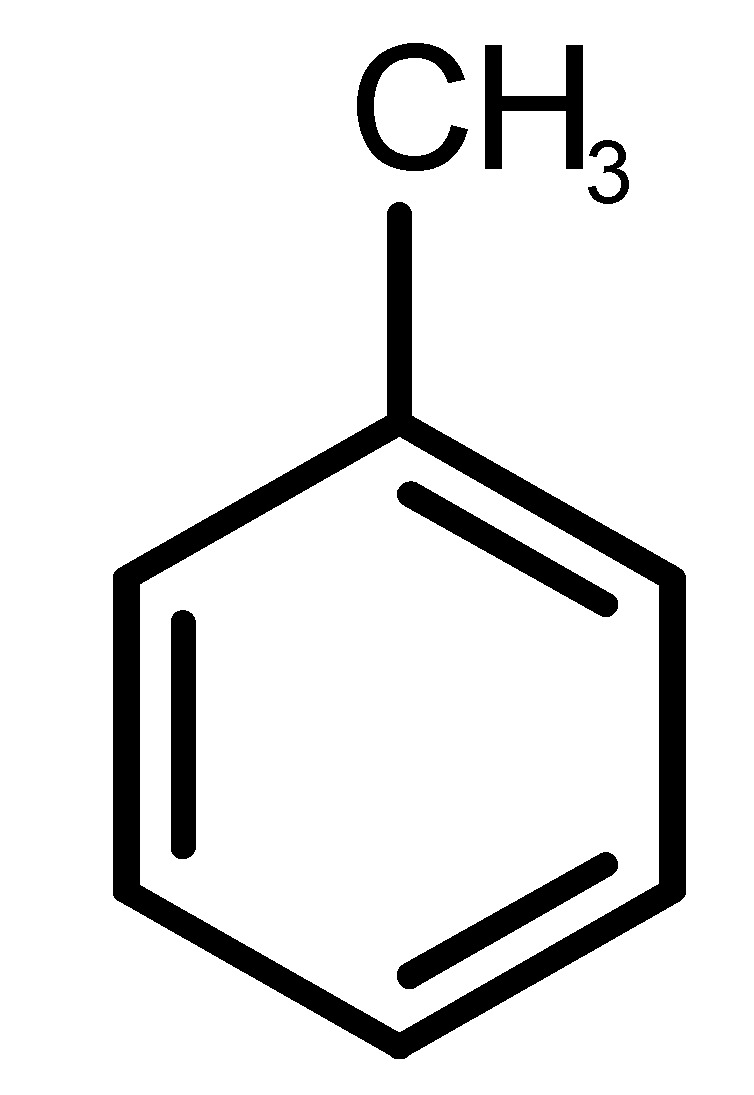	*toluene* (paint-thinner/pungent)	92.14	2.73	28.400	3	4	−5.7
60	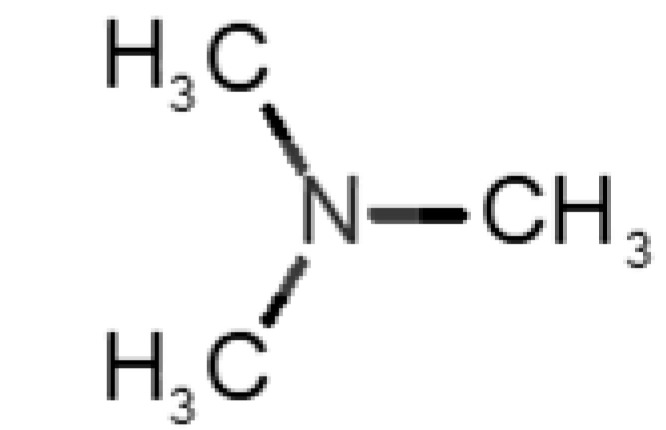	*trimethylamine* (fishy/ammoniacal)	59.11	0.16	1610	0	0	−2.0

## Data Availability

Not applicable.

## Supplementary Materials

The following are available online at https://www.mdpi.com/2218-273X/11/2/145/s1. Odorant molecules CAS identification are listed in Table S1. Statistical analysis on the comparison of odorant properties (described in Table 1 and Table 2) in Table S2. The Vina grid box in Figure S1, the X-ray and Uniprot hOBP sequences alignment are presented in Figure S2 and the RMSD and cartoon structural superposition in Figures S3 and S4, respectively. Standard scatter plots/linear correlation regression are presented as SM, in Figures S5–S9, evaluating the relation of ΔG_binding_ to the other properties separately. The docking binding energies against the 4RUN X-ray structure are shown in Table S3.

## Abbreviations

hOBP	Human odorant-binding protein
PCA	Principal component analysis
OR	Olfactory receptor
MD	Molecular dynamics
MW	Molecular weight
Vp	Vapor pressure (volatility)
logP	Partition coefficient (hydrophobicity level)
N°DB	Number of double bonds
DoU	Degree of unsaturation
