# Supplementary material for: The Structural Properties of Odorants Modulate Their Association to Human Odorant Binding Protein
Source: Biomolecules. 2021 Jan 22;11(2):145. doi: 10.3390/biom11020145 (PMC7912024; doi:10.3390/biom11020145)
Supplement: Supplementary file 1 [file biomolecules-11-00145-s001.pdf]

biomolecules

Supplementary materials

The Structural Properties of Odorants Modulate Their Association to Human Odorant Binding Protein

Tarsila G. Castro, Carla Silva, Teresa Matamá and Artur Cavaco-Paulo *

Centre of Biological Engineering, University of Minho, Campus de Gualtar, 4710-057 Braga, Portugal
* Correspondence: artur@deb.uminho.pt; Tel.: +351-253-604-409

Citation: Castro, T.G.; Silva, C.; Matamá, T.; Cavaco-Paulo, A. The Structural Properties of Odorants Modulate Their Association to Human Odorant Binding Protein. **2021**, *11*, 145. https://doi.org/10.3390/biom11020145

Received: 24 November 2020
Accepted: 20 January 2021
Published: 22 January 2021

Publisher's Note: MDPI stays neutral with regard to jurisdictional claims in published maps and institutional affiliations.

Copyright: © 2021 by the authors. Submitted for possible open access publication under the terms and conditions of the Creative Commons Attribution (CC BY) license (http://creativecommons.org/licenses/by/4.0/).

Table S1. CAS# = chemical abstract service, identification, of each odorant molecule under study.

	CAS#		CAS#
**1**	85213-22-5	**31**	4312-99-6
**2**	8000-41-7	**32**	2463-53-8
**3**	502-99-8	**33**	58888-76-9
**4**	140-11-4	**34**	27960-21-0
**5**	123-86-4	**35**	307964-33-6
**6**	76-22-2	**36**	64-19-7
**7**	6485-40-1	**37**	539-86-6
**8**	5392-40-5	**38**	870-23-5
**9**	106-22-9	**39**	7664-41-7
**10**	91-64-5	**40**	62-53-3
**11**	431-03-8	**41**	100-53-8
**12**	97-53-0	**42**	109-79-5
**13**	6413-10-1	**43**	462-94-2
**14**	706-14-9	**44**	7782-50-5
**15**	104-61-0	**45**	67-66-3
**16**	106-24-1	**46**	676-59-5
**17**	24851-98-7	**47**	7783-06-4
**18**	123-92-2	**48**	120-72-9
**19**	67920-63-2	**49**	503-74-2
**20**	5989-27-5	**50**	74-93-1
**21**	126-91-0	**51**	593-54-4
**22**	55066-48-3	**52**	7697-37-2
**23**	2216-51-5	**53**	10102-44-0
**24**	623-42-7	**54**	7803-51-2
**25**	123-35-3	**55**	79-09-4
**26**	80-56-8	**56**	110-60-1
**27**	357650-26-1	**57**	83-34-1
**28**	89-82-7	**58**	4756-05-2
**29**	65113-99-7	**59**	108-88-3
**30**	121-33-5	**60**	75-50-3

Table S2. Comparison of the two independent groups of selected pleasant and unpleasant odorant molecules regarding the six physicochemical properties under study. Results of the nonparametric Mann-Whitney U-test procedure for each property are presented for a level of significance of 0.05.

	**Pleasant odorants**			**Unpleasant odorants**			**Mann-Whitney U test results**		
	**Median**	**Sum of ranks**	**n**	**Median**	**Sum of ranks**	n	**U**	*p* value	ES (|z|/√N)
**MW**	**152,24**	1281,5	30	**89,165**	548,5	30	83,5	$p < 0.0001$	0,70
**logP**	**2,69**	1197	30	**0,875**	633	30	168	$p < 0.0001$	0,54
**Vp**	**0,1075**	676	30	**22,065**	1154	30	211	$p < 0.001$	0,46
**NºDB**	**2**	1115	30	**1**	715	30	250	$p < 0.01$	0,38
**DoU**	**3**	1174	30	**0,75**	656	30	191	$p < 0.001$	0,49
**$\Delta G_{binding}$**	**-5,75**	579	30	**-3,15**	1251	30	114	$p < 0 .0001$	0,64

Table S3. $\Delta G_{binding}$ (kcal/mol) of the 60 odorants molecules complexed to hOPB from **4RUN X-ray** structure. Comparing the ranked $\Delta G_{binding}$ values obtained for 4RUN structure, here described, with the ones obtained for the MD simulation of hOBP, either for the pleasant group of odorants (Table 1) or the unpleasant group (Table2), the Mann-Whitney U test shows no significant difference between the two structures (U= 423, n_1=30, n_2 = 30, p>0.05 for the pleasant group of odorants; U= 422.5, n_1=30, n_2 = 30, p>0.05 for the unpleasant group of odorants).

	**$\Delta G_{binding}$ (kcal/mol)**		**$\Delta G_{binding}$ (kcal/mol)**
**1**	-4,7	**31**	-4,8
**2**	-5,5	**32**	-5,5
**3**	-5,8	**33**	-4,6
**4**	-5,9	**34**	-5,1
**5**	-4,3	**35**	-4,3
**6**	-6,7	**36**	-3,3
**7**	-6,0	**37**	-3,9
**8**	-5,8	**38**	-2,9
**9**	-5,7	**39**	-1,3
**10**	-6,8	**40**	-4,7
**11**	-4,1	**41**	-4,8
**12**	-5,9	**42**	-3,4
**13**	-4,8	**43**	-4,1
**14**	-5,5	**44**	-2,1
**15**	-5,4	**45**	-3,3
**16**	-5,7	**46**	-1,9
**17**	-5,9	**47**	-0,8
**18**	-4,9	**48**	-5,7
**19**	-5,6	**49**	-4,7
**20**	-5,5	**50**	-1,3
**21**	-5,2	**51**	-1,4
**22**	-6,1	**52**	-3,1
**23**	-5,0	**53**	-2,9
**24**	-4,3	**54**	-0,8
**25**	-5,7	**55**	-3,9
**26**	-6,3	**56**	-3,8
**27**	-5,7	**57**	-5,7
**28**	-5,5	**58**	-3
**29**	-6,1	**59**	-5,1
**30**	-5,5	**60**	-2,5

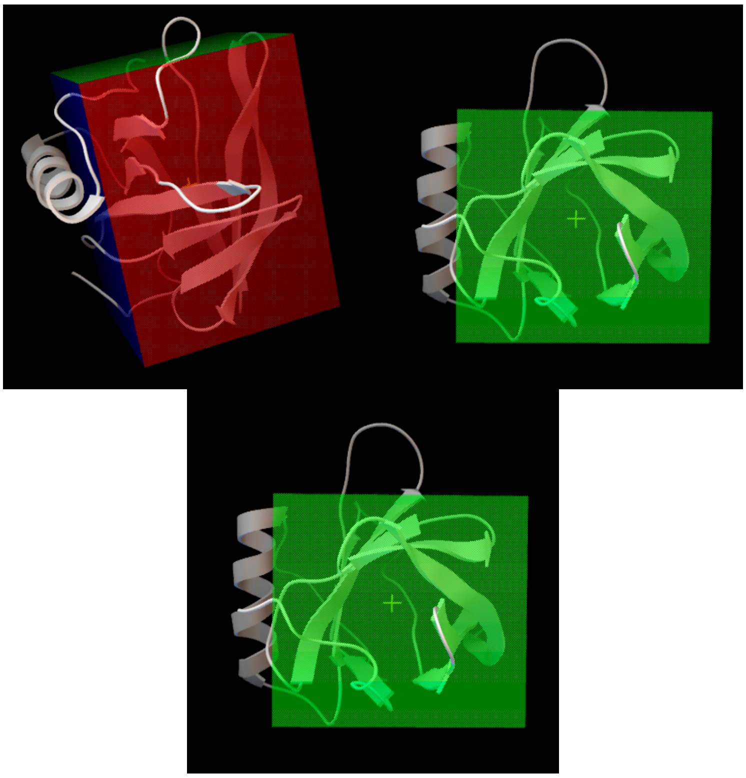

Figure S1. Snapshot of Vina grid box, in different perspectives, encompassing the entire beta barrel and its extremities (loops and bends). .

```
CLUSTAL O(1.2.4) multiple sequence alignment

4RUN_1|Chains               -------EDITGTWYVKAMVVDKDFPEDRRPRKVSPVKVTALGGGNLEATFTFMREDRCI 53
sp|Q9NY56|OBP2A_HUMAN       LSFTLEEEDITGTWYVKAMVVDKDFPEDRRPRKVSPVKVTALGGGNLEATFTFMREDRCI 60
                                   *****************************************************

4RUN_1|Chains               QKKILMRKTEEPGKFSAYGGRKLIYLQELPGTDDYVFYSKDQRRGGLRYMGNLVGRNPNT 113
sp|Q9NY56|OBP2A_HUMAN       QKKILMRKTEEPGKFSAYGGRKLIYLQELPGTDDYVFYCKDQRRGGLRYMGKLVGRNPNT 120
                            **************************************.************:********

4RUN_1|Chains               NLEALEEFKKLVQHKGLSEEDIFMPLQTGSCVLEHHHHHHH    154
sp|Q9NY56|OBP2A_HUMAN       NLEALEEFKKLVQHKGLSEEDIFMPLQTGSCVLEH------    155
                            ***********************************
```

Figure S2. Alignment from Clustal Omega (https://www.ebi.ac.uk/Tools/msa/clustalo/) between hOBP 4RUN X-ray structure and the full canonical sequence in UniProt.

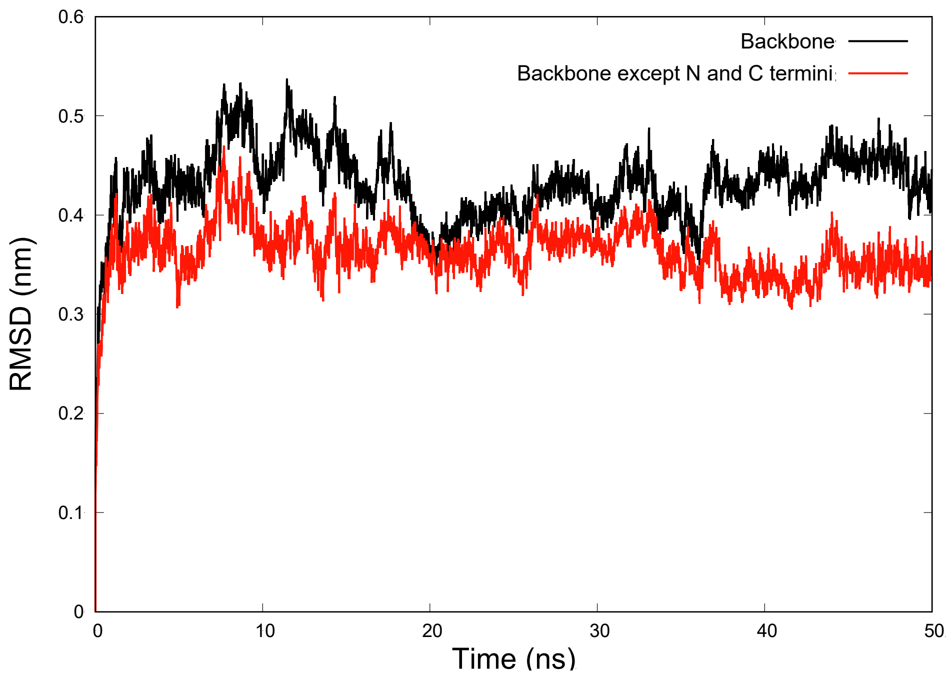

Figure S3. Backbone Root Mean Square Deviation (RMSD) of 50 ns MD simulation of hOBP. Black trace considering the backbone of all amino acid residues and red trace disregarding the N and C termini.

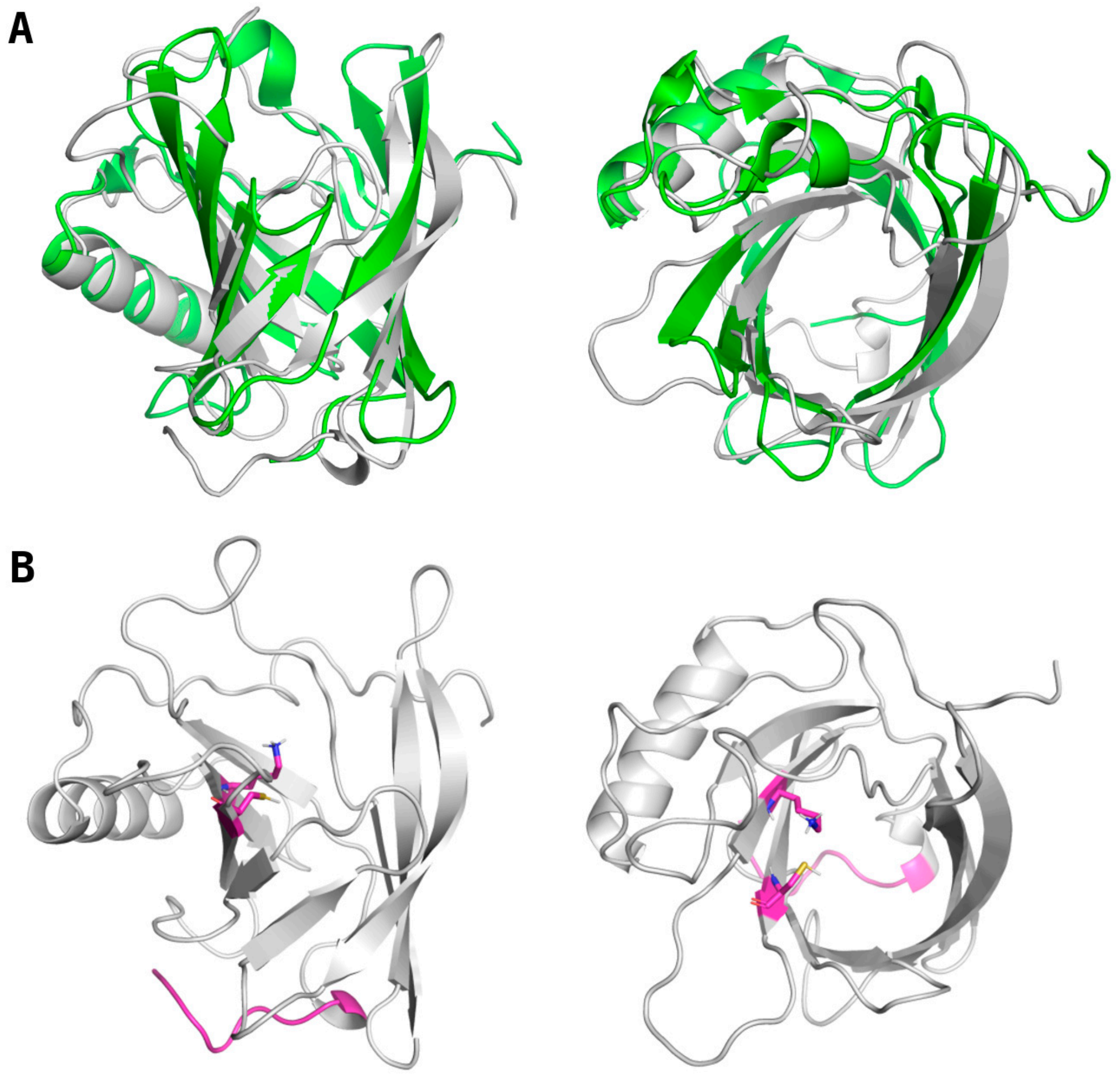

Figure S4. (A) Cartoon alignment superposition of 4RUN X-ray structure, in green, over modelled middle structure, in grey. (B) Middle structure, from MD simulations, highlighting in magenta the modelled N-terminal portion and the 2 distinct amino acids, at the barrel core, which in this most frequent conformation have the side chains facing the interior.

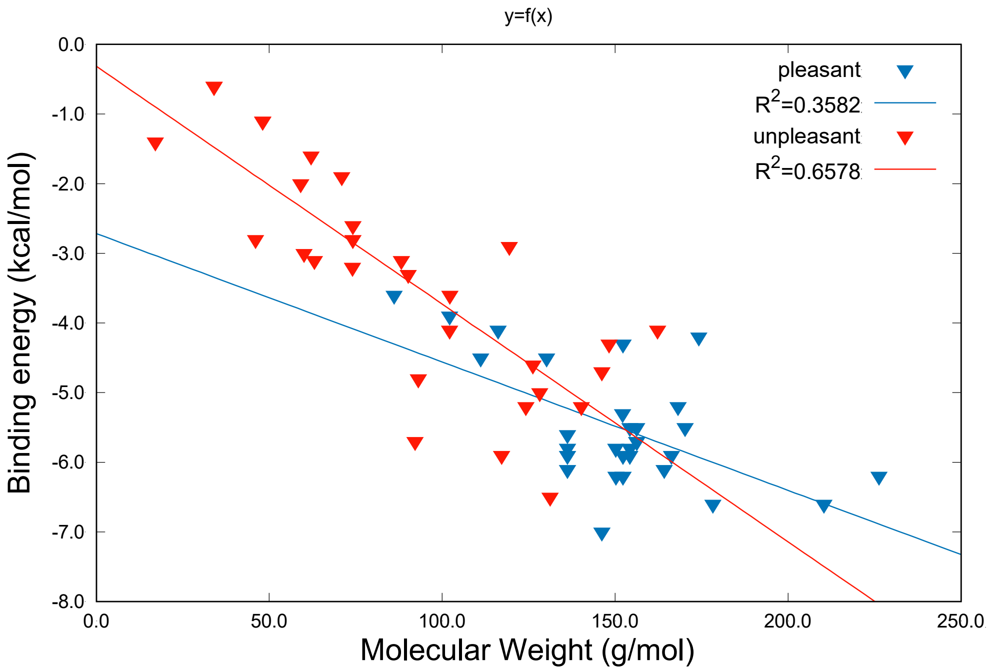

Figure S5. Scatter plot of binding energy versus MW, considering the 30 pleasant and the 30 unpleasant odorant molecules. The solid line represents the regression line calculated for the linear correlation between the variables.

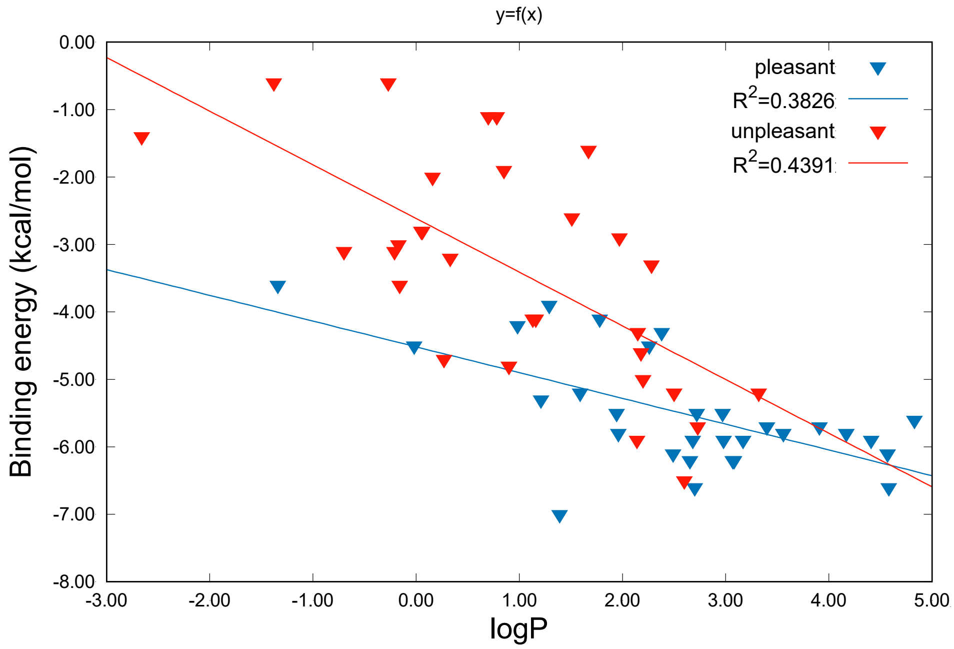

Figure S6. Scatter plot of binding energy versus logP, considering the 30 pleasant and the 30 unpleasant odorant molecules. The solid line represents the regression line calculated for the linear correlation between the variables.

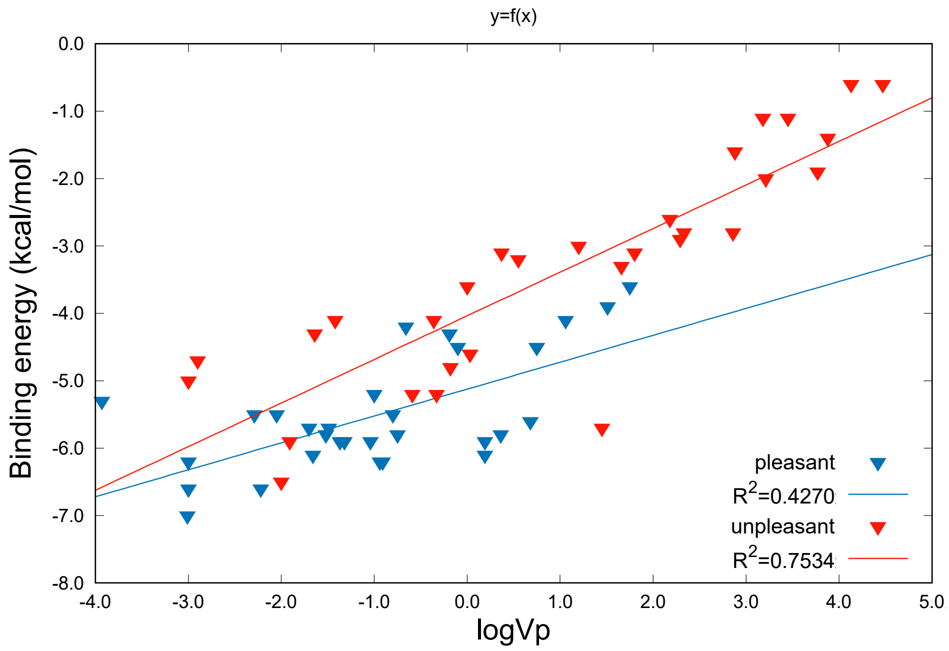

Figure S7. Scatter plot of binding energy versus logVp, considering the 30 pleasant and the 30 unpleasant odorant molecules. The solid line represents the regression line calculated for the linear correlation between the variables.

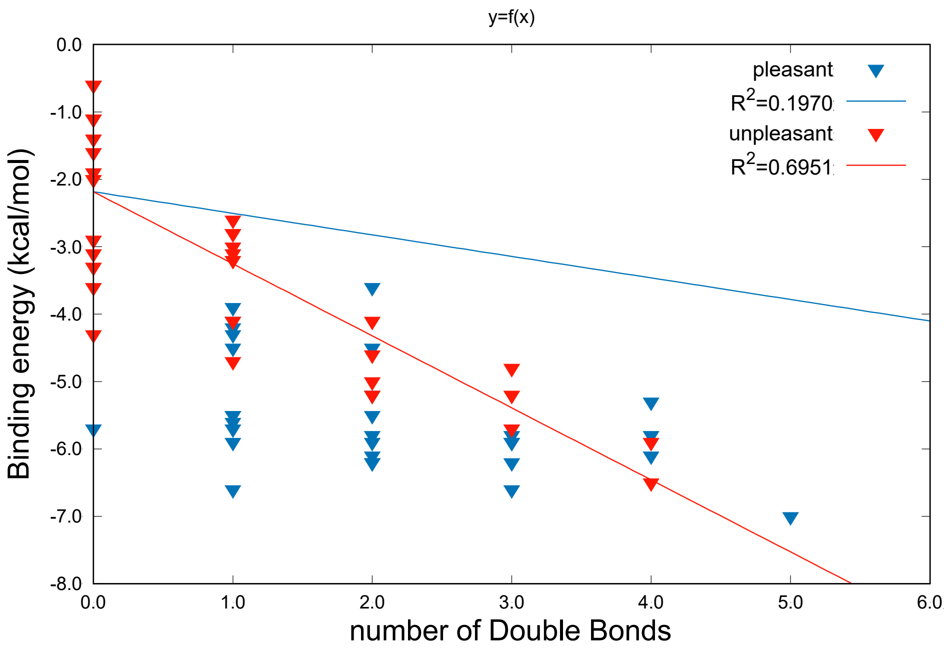

Figure S8. Scatter plot of binding energy versus nºDB, considering the 30 pleasant and the 30 unpleasant odorant molecules. The solid line represents the regression line calculated for the polynomial correlation between the variables.

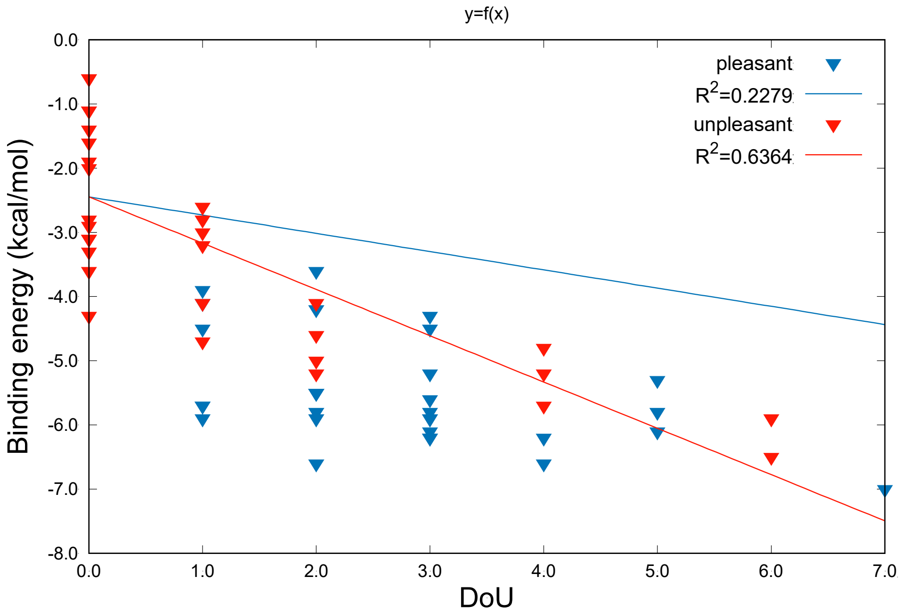

Figure S9. Scatter plot of binding energy versus Degree of Unsaturation, considering the 30 pleasant and the 30 unpleasant odorant molecules. The solid line represents the regression line calculated for the polynomial correlation between the variables.
